# Mulberry Biomass-Derived Nanomedicines Mitigate Colitis through Improved Inflamed Mucosa Accumulation and Intestinal Microenvironment Modulation

**DOI:** 10.34133/research.0188

**Published:** 2023-07-07

**Authors:** Wenjing Yang, Ya Ma, Haiting Xu, Zhenhua Zhu, Jiaxue Wu, Cheng Xu, Wei Sun, Erhu Zhao, Min Wang, Rui L. Reis, Subhas C. Kundu, Xiaoxiao Shi, Bo Xiao

**Affiliations:** ^1^ State Key Laboratory of Silkworm Genome Biology, College of Sericulture, Textile, and Biomass Sciences, Southwest University, Chongqing 400715, China.; ^2^ Chongqing Key Laboratory of Soft-Matter Material Chemistry and Function Manufacturing, Faculty of Materials and Energy, Southwest University, Chongqing 400715, China.; ^3^ Department of Gastroenterology, The First Affiliated Hospital of Nanchang University, Nanchang 330006, China.; ^4^ 3Bs Research Group, I3Bs — Research Institute on Biomaterials, Biodegradables and Biomimetics, University of Minho, Headquarters of the European Institute of Excellence on Tissue Engineering and Regenerative Medicine, AvePark, Barco 4805-017, Guimaraes, Portugal.; ^5^ ICVS/3B’s-PT Government Associate Laboratory, Braga, Guimarães, Portugal.

## Abstract

The therapeutic outcomes of conventional oral medications against ulcerative colitis (UC) are restricted by inefficient drug delivery to the colitis mucosa and weak capacity to modulate the inflammatory microenvironment. Herein, a fluorinated pluronic (FP127) was synthesized and employed to functionalize the surface of mulberry leaf-derived nanoparticles (MLNs) loading with resveratrol nanocrystals (RNs). The obtained FP127@RN-MLNs possessed exosome-like morphologies, desirable particle sizes (around 171.4 nm), and negatively charged surfaces (−14.8 mV). The introduction of FP127 to RN-MLNs greatly improved their stability in the colon and promoted their mucus infiltration and mucosal penetration capacities due to the unique fluorine effect. These MLNs could efficiently be internalized by colon epithelial cells and macrophages, reconstruct disrupted epithelial barriers, alleviate oxidative stress, provoke macrophage polarization to M2 phenotype, and down-regulate inflammatory responses. Importantly, in vivo studies based on chronic and acute UC mouse models demonstrated that oral administration of chitosan/alginate hydrogel-embedding FP127@RN-MLNs achieved substantially improved therapeutic efficacies compared with nonfluorinated MLNs and a first-line UC drug (dexamethasone), as evidenced by decreased colonic and systemic inflammation, integrated colonic tight junctions, and intestinal microbiota balance. This study brings new insights into the facile construction of a natural, versatile nanoplatform for oral treatment of UC without adverse effects.

## Introduction

Ulcerative colitis (UC) is a chronic and recurrent inflammatory disorder in the colon, whose morbidity increased steadily worldwide in recent decades [1]. Existing studies are deemed to stem from increased oxidative stress, impaired colonic epithelial barrier, persistent mucosal inflammation, and microbiota dysbiosis [2]. Current clinical medications, such as steroids, immunomodulators, and biological drugs, can partially relieve UC symptoms mainly through the suppression of inflammatory reactions [3]. Still, their long-term utilities lead to unsatisfactory therapeutic outcomes, drug resistance, and serious adverse effects (e.g., hepatotoxicity, infections, and cancers) [4]. Therefore, the development of novel therapeutic regimens is urgently required for the treatment of UC.

The oral route is the most widely used approach for drug administration due to its high patient compliance, relative safe, cost-effectiveness, and direct drug delivery to the colitis mucosa [5]. However, oral drug delivery to the colon tissues has been rendered incredibly challenging owing to numerous anatomical and physiological obstacles in the gastrointestinal tract (GIT), including gastric acid, digestive enzymes, mucus layer, and epithelial barrier [6]. Recently, we and other groups reported that natural exosome-like nanotherapeutics could be massively extracted from diverse edible plant (e.g., tea leaf, grape, and ginger), which presented excellent biosafety and maintained stability during their passage through the GIT [7,8]. Nevertheless, they could accumulate in the colon tissues and be selectively internalized by activated macrophages via galactose receptor-mediated endocytosis, achieving preventive and therapeutic effects against colon diseases [7]. Therefore, they have been brought into the clinic for treating irritable bowel disease and colon cancer (https://clinicaltrials.gov; NCT04879810 and NCT01294072).

Despite the promising clinical application of edible plant-extracted nanotherapeutics, they are still undergoing several translational barriers, mainly including the following 6 aspects: (a) hard to massive production, (b) cold chain storage (usually −80.0 °C), (c) low contents of bioactive components, (d) lack of mucus penetration capacity, (e) limited mucosa accumulation, and (f) inefficient cellular uptake [8–10]. To address the first 3 barriers (a, b, and c), lipids were extracted from edible plants on a large scale and further encapsulated exogenous drugs to attain the freeze-dried nanomedicines [11]. Colonic mucus (~830 μm) is a highly adhesive and viscoelastic network that effectively capture and eliminate foreign matters via intestinal peristalsis and mucin turnover [12]. Therefore, the mucus layer has been regarded as the toughest barrier for oral drug delivery to the underlying colonic mucosa [13,14]. It was reported that the surface decoration of nanoparticles (NPs) with poly(ethylene glycol), poly(2-alkyl-2-oxazolines), and polyzwitterionic polymer facilitated their mucus penetration through the decreases of hydrogen bonding and hydrophobic interactions with glycomucins [15]; however, these molecules also hindered the cellular uptake of NPs [16]. Subsequent studies led by our group as well as others demonstrated that pluronic F127 (P127), a Food and Drug Administration-approved polymer, could not only improve the mucus penetration capacity but also increase the cell internalization efficiency of NPs [17,18]. A recent investigation revealed that fluoroalkylation could enhance the physiological stability, resist the adsorption of surrounding proteins, and improve the bladder mucus penetration of polymeric NPs due to the fluorination effect [19,20]. Given these attractive properties of fluoroalkylation, we hypothesize that the introduction of fluorocarbon to P127 (FP127) would overcome the drug delivery barriers (a, b, and c) of edible plant-derived nanomedicines in the GIT, leading to the improved therapeutic outcomes against UC.

**Fig. 1.**
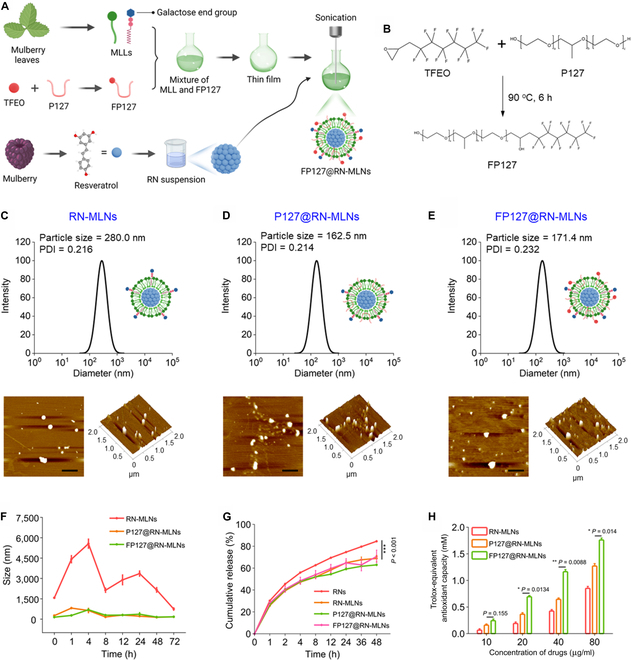
Fabrication and physicochemical characterization of FP127@RN-MLNs. (A) Schematic diagram showing the procedures for fabricating FP127@RN-MLNs. (B) Synthetic route of FP127. Particle size distributions and AFM images of (C) RN-MLNs, (D) P127@RN-MLNs, and (E) FP127@RN-MLNs. Scale bar = 400 nm. (F) Variations of hydrodynamic particle sizes of RN-MLNs, P127@RN-MLNs, and FP127@RN-MLNs during incubation in the colonic simulation solutions (pH = 6.8) for 72 h. Data are expressed as means ± SEM (*n* = 3). (G) Cumulative drug release behaviors of RNs, RN-MLNs, P127@RN-MLNs, and FP127@RN-MLNs in the colonic simulation solutions (pH = 6.8). (H) Antioxidant activities of RN-MLNs, P127@RN-MLNs, and FP127@RN-MLNs at different resveratrol concentrations. Data are expressed as means ± SEM (*n* = 3). PDI, polydispersity index.

**Fig. 2.**
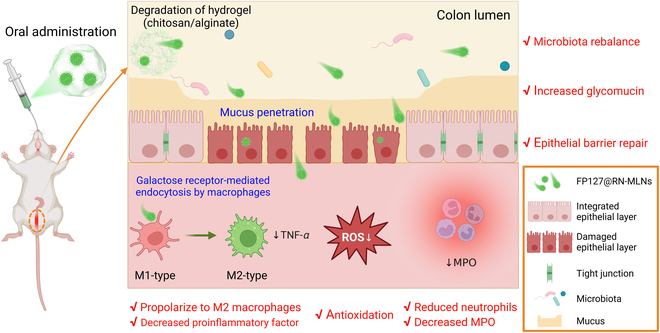
Schematic diagram of intestinal passage and therapeutic mechanism of hydrogel-embedding FP127@RN-MLNs against UC after oral administration. Once FP127@RN-MLN-embedded hydrogel reaches the colon, the chitosan/alginate hydrogel is disrupted, and FP127@RN-MLNs are released from the hydrogel into the colonic lumen. Subsequently, these MLNs penetrate through the mucus layer and are internalized by colon epithelial cells and activated macrophages via galactose receptor-mediated endocytosis. Finally, they exert the therapeutic outcomes against UC through epithelial barrier repair, antioxidation, anti-inflammation, increased glycomucin, and microbiota rebalance.

To verify this hypothesis, we selected resveratrol as a model drug [21–23], processed it into nanocrystals (RNs), and further loaded them into FP127-functionalized mulberry leaf lipid (MLL)-based NPs (MLNs) to obtain multifunctional therapeutic regimens (FP127@RN-MLNs) (Fig. 1A). After oral administration, FP127@RN-MLNs could remain stable during their passage through the upper GIT and be released explicitly to the colonic lumen assisted by a chitosan/alginate hydrogel. Thereafter, these MLNs penetrated through the mucus layer and the disrupted epithelial barrier in the presence of FP127 and enhanced permeation and retention effect. They were internalized by the colonic epithelia and macrophages because of the end galactose groups in the MLLs. Eventually, they exerted therapeutic effects against UC through promigration of colonic epithelium to the wound, free radical elimination, down-regulation of proinflammatory cytokines, propolarization of macrophages to M2 type, and intestinal microbiota rebalance (Fig. 2).

## Results

### Construction and physicochemical properties of MLNs

In an alkaline environment, the hydroxyl groups of P127 reacted with the epoxy groups of tridecafluoroheptyl ethylene oxide (TFEO) using triethylenediamine as a catalytic agent, and the fluorinated product (FP127) was obtained during this nucleophilic substitution process (Fig. 1B). ^1^H nuclear magnetic resonance spectra revealed that following the reaction with TFEO, FP127 appeared at characteristic peaks H-1 and H-2 at chemical shifts 5.33 (peak a) and 2.01 ppm (peak b), and the substitution rate of the functional group in P127 was about 46.5% (Fig. S1). Additionally, the area of peak b was equal to approximately twice as large as peak a, indicating that the number of hydrogen atoms at compound b doubled that at a, in line with the chemical structural formula of FP127. Prior to the preparation of RNs, resveratrol was fully dissolved in ethanol to form an organic phase, and polyvinylpyrrolidone was dissolved in deionized water to form an aqueous phase. Upon rapidly adding this organic phase to the aqueous phase, resveratrol nanocrystals (RNs) were generated by crystalline precipitation due to the low aqueous solubility of resveratrol. Three types of MLL-based NPs (namely, RN-MLNs, P127@RN-MLNs, and FP127@RN-MLNs) were fabricated by the thin-film hydration method. During the formation of FP127@RN-MLNs, the hydrophobic sections of FP127 (poly(phenylene oxide blocks) penetrated into the phospholipid bilayers, while the hydrophilic segments (poly(ethylene oxide blocks) and hydro-oleophobic segments (perfluorocarbons) of FP127 surrounded MLNs and stabilized them due to steric hindrance.

The particle sizes and zeta potentials of MLNs were determined by dynamic light scattering. RNs possessed a diameter of 86.8 nm and a negative-charged surface of −18.0 mV. After surface coating, the particle sizes of RN-MLNs, P127@RN-MLNs, and FP127@RN-MLNs increased to 280.0, 162.5, and 171.4 nm, respectively, and the polydispersity indexes of all these MLNs were less than 0.3, indicating their uniform size distributions. Compared with the particle sizes of RN-MLNs, P127@RN-MLNs and FP127@RN-MLNs showed smaller particle sizes, and this phenomenon could be attributed to the presence of P127 or FP127 on their surfaces, which avoided the aggregation of MLNs in aqueous suspensions. Furthermore, the morphological characteristics of these MLNs were detected by atomic force microscopy (AFM). It was observed that all MLNs were spherical, and their particle sizes were lower than 100 nm (Fig. 1C to E). The particle sizes of MLNs obtained from AFM were much smaller than those determined by dynamic light scattering, which could be attributed to the dehydration and shrinkage of MLNs during AFM examinations.

To evaluate the stabilities of MLNs in the colon lumen, their variations in particle sizes and surface charges were studied in simulated colonic buffers (pH = 6.8). Even though the surface charges of RN-MLNs maintained stable, their particle sizes increased sharply during the initial 4-h incubation in the colonic simulation solutions (pH = 6.8), possibly due to the aggregation of these MLNs (Fig. 1F and Fig. S2). Thereafter, their particle sizes gradually decreased, which could be ascribed to the disintegration of MLN aggregates in a shaker. However, P127@RN-MLNs and FP127@RN-MLNs showed quite stable particle sizes, suggesting that P127 and FP127 could prevent the aggregation of MLNs and endow them with excellent stabilities in the colon lumen. Since controlled drug release to the colitis tissues is essential for UC treatment, we studied the resveratrol release profiles. Figure 1G showed that all samples presented constant drug release behaviors. The resveratrol release rate of RNs was markedly faster than those of MLL-coated MLNs, which was attributed to the surface coating of RNs with MLLs. During UC development, activated macrophages produce excessive amounts of reactive oxygen species (ROS) [24], aggravating mucosal damage and inflammatory reactions in the colonic region. To investigate whether MLNs could address the adverse effects of ROS, we determined their total antioxidant capacity using a 2, 2′-azino-di-(3-ethylbenzthiazoline sulfonate) assay kit. Figure 1H revealed that the antioxidant properties of these MLNs increased as the resveratrol concentrations increase, and FP127@RN-MLNs showed superior antioxidant activity when compared with RN-MLNs and P127@RN-MLNs.

### FP127 increases uptake efficiency of MLNs by CT-26 cells and improves cell migration

The excellent biocompatibility of nanomedicines is a requisite for their medical applications. Colonic epithelial cells and macrophages are 2 target cells in treating UC [25,26], and accordingly, their 2 representatives (CT-26 cells and Raw 264.7 macrophages) were selected, against which we evaluated the in vitro cytotoxicities of MLNs using tetrazolium assay. As seen in Fig. S3, the viabilities of both cell lines receiving the treatment of RN-MLNs, P127@RN-MLNs, and FP127@RN-MLNs were over 85.2% after coincubation for 24 h, suggesting their good in vitro biosafety.

**Fig. 3.**
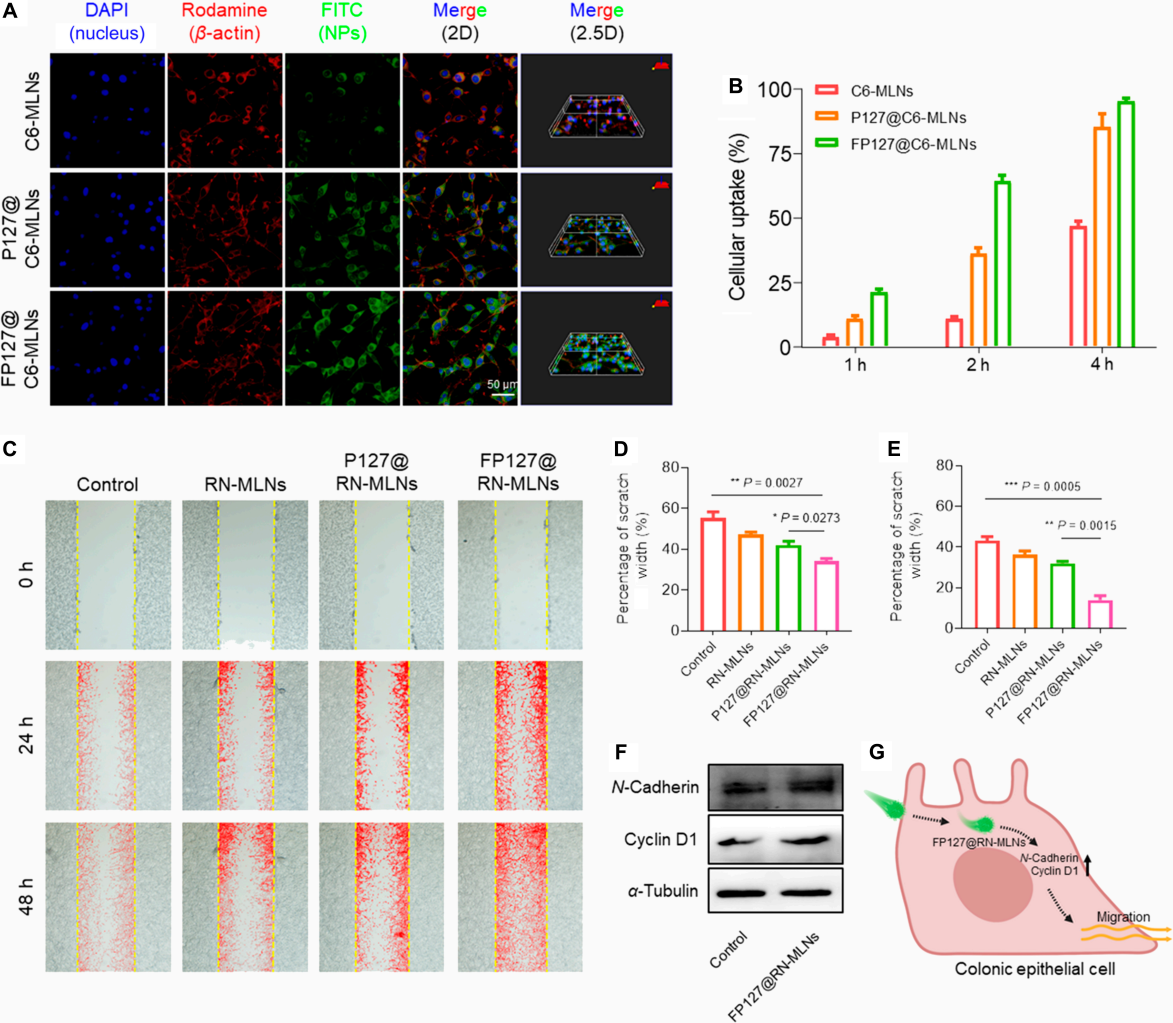
Cell internalization profiles of MLNs by CT-26 cells and wound healing capacities of various MLNs. (A) Fluorescence images of CT-26 cells with the treatment of C6-MLNs, P127@C6-MLNs, and FP127@C6-MLNs for 4 h. Scale bar = 50 μm. (B) Quantification of cellular uptake percentages of C6-MLNs, P127@C6-MLNs, and FP127@C6-MLNs by CT-26 cells after coincubation for 1, 2, and 4 h, respectively. Data are expressed as means ± SEM (*n* = 3). (C) Scratch images of the CT-26 cell layers with the treatment of RN-MLNs, P127@RN-MLNs, and FP127@RN-MLNs for 24 and 48 h, respectively. Quantitative analysis of the scratched widths for CT-26 cell layers after the treatment with RN-MLNs, P127@RN-MLNs, and FP127@RN-MLNs for (D) 24 and (E) 48 h, respectively. Data are expressed as means ± SEM (*n* = 3). (F) Expression profiles of *N*-Cadherin and Cyclin D1 proteins in CT-26 cells with the treatment of FP127@RN-MLNs for 24 h. *α*-Tubulin was selected as a control. (G) Schematic diagram of the promigration mechanism of FP127@RN-MLNs. DAPI, 4′, 6-diamidino-2-phenylindole; FITC, fluorescein isothiocyanate.

Resveratrol is known to exert its therapeutic effects mainly within cells, and thus, it becomes necessary to internalize resveratrol-loaded MLNs by the target cells. To track the in vitro biodistribution of MLNs, coumarin-6 (C6) was used as a fluorescent probe. The cellular uptake efficiencies of various MLNs were comparatively investigated by confocal laser scanning microscopy and flow cytometry. Figure 3A presented that C6-MLN-treated cells displayed weaker green fluorescence than CT-26 cells with the treatment of P127@C6-MLNs and FP127@C6-MLNs. We further found that the uptake percentages of MLNs by CT-26 cells changed in an incubation time-dependent manner. The FP127@C6-MLN-treated cells showed the highest cellular uptake percentage among all the treatment groups (Fig. 3B). In particular, 2 h after coincubation, the cell internalization percentage of FP127@C6-MLNs was 5.8- and 1.7-fold higher than those of C6-MLNs and P127@C6-MLNs, respectively. It was worth noting that over 95% of CT-26 cells took up FP127@C6-MLNs after incubation for 4 h. The fluorescence images and mean fluorescence intensity (MFI) results confirmed the synergistically improved cellular uptake effects of P127 and fluoroalkane (Figs. S4 and S5), which might be ascribed to the enhanced interaction between MLNs and cell membranes.

The colonic epithelial layer serves as the first barrier for the underlying mucosa, which is, unfortunately, prone to being disrupted during the development of UC. This causes the invasion of pathogenic microorganisms and foreign matters into the colon tissues, leading to aggravating UC symptoms [27]. Therefore, mucosal restoration was one of the most essential therapeutic aims for UC treatment [28]. Considering that cell migration is a critical step during epithelial wound healing, we assessed the improved migration of CT-26 cells by various MLNs using the scratch-wound assay. As revealed in Fig. 3C, the scratch widths of all the experimental groups decreased as incubation time increased. Twenty-four hours after coincubation, the FP127@RN-MLN-treated cells showed the narrowest scratch area (Fig. 3D). After 48 h, the control group showed the largest cell scratch width among all the groups, and the scratch width in the cell layers receiving the treatment of RN-MLNs, P127@RN-MLNs, and FP127@RN-MLNs decreased to 36.4%, 32.1%, and 13.9%, respectively (Fig. 3E). As reported, high expression levels of *N*-Cadherin and Cyclin D1 proteins can activate migration-related signaling pathways [29]. Western blot results indicated that the expression levels of these 2 proteins were up-regulated in the FP127@RN-MLN-treated cells, compared with those in the control group (Fig. 3F), thereby promoting the migration of CT-26 cells to the wound surface and facilitating wound healing (Fig. 3G).

### FP127 increases macrophage uptake efficiency and anti-inflammatory activity of MLNs

**Fig. 4.**
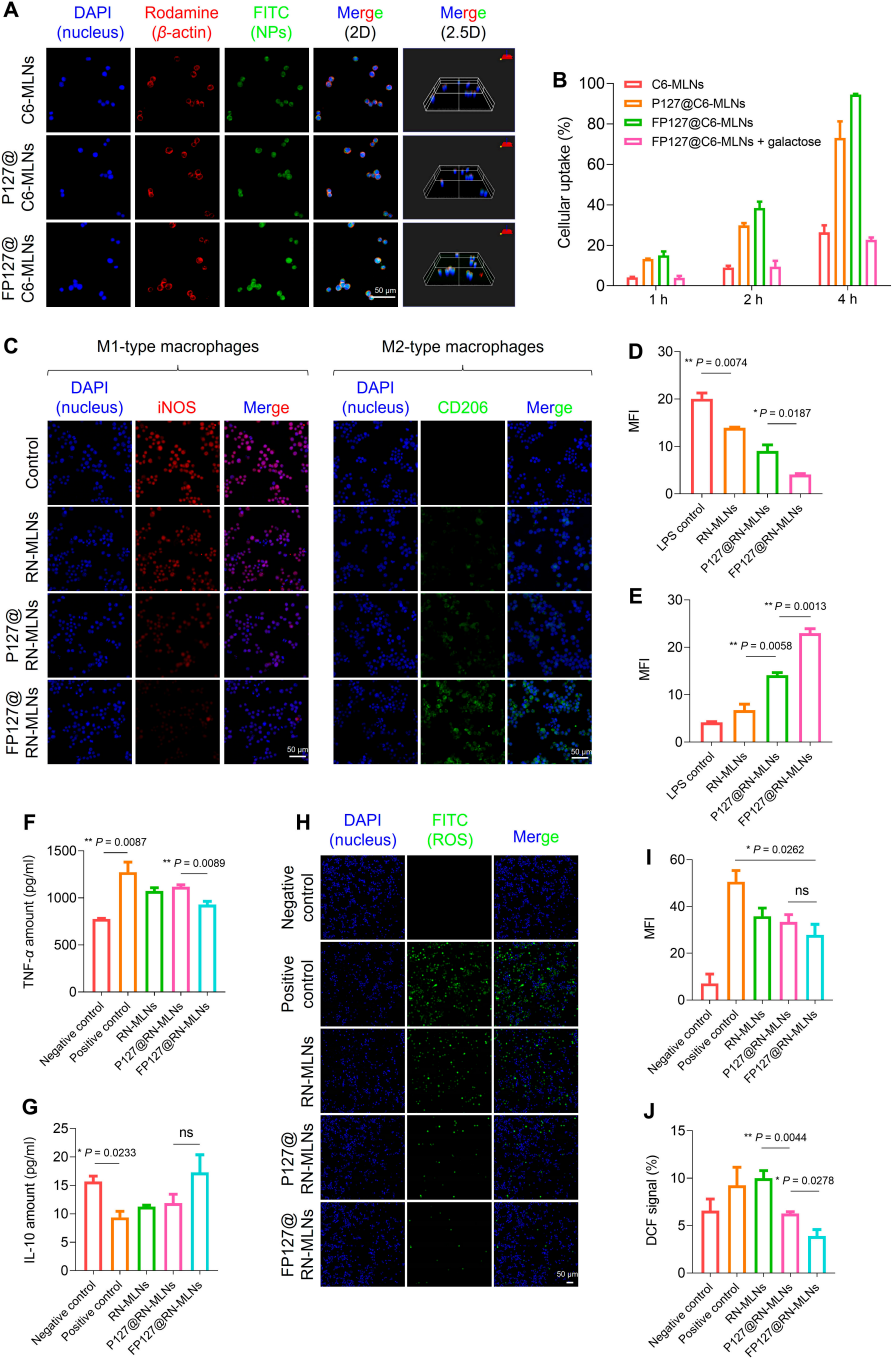
In vitro macrophage uptake profiles, anti-inflammatory activities, and antioxidant properties of various MLNs. (A) Fluorescence images of Raw 264.7 macrophages with the treatment of C6-MLNs, P127@C6-MLNs, and FP127@C6-MLNs for 4 h. Scale bar = 50 μm. (B) Quantification of cellular uptake profiles of C6-MLNs, P127@C6-MLNs, and FP127@C6-MLNs by Raw 264.7 macrophages at different time points (1, 2, and 4 h). Data are expressed as means ± SEM (*n* = 3). (C) Immunofluorescence staining images of iNOS (the typical marker for M1-type macrophage) and CD206 (the typical marker for M2-type macrophage) in Raw 264.7 macrophages with the treatment of RN-MLNs, P127@RN-MLNs, and FP127@RN-MLNs for 24 h. Scale bar = 50 μm. Quantification of the relative fluorescence intensities of (D) iNOS and (E) CD206 in Raw 264.7 macrophages with the treatment of RN-MLNs, P127@RN-MLNs, and FP127@RN-MLNs for 24 h. Levels of (F) TNF-*α* and (G) IL-10 secreted from Raw 264.7 macrophages with the treatment of RN-MLNs, P127@RN-MLNs, and FP127@RN-MLNs for 24 h. (H) In vitro antioxidant activities of RN-MLNs, P127@RN-MLNs, and FP127@RN-MLNs after the incubation with Raw 264.7 macrophages for 24 h. Scale bar = 50 μm. (I) Quantification of the relative fluorescence intensity of intracellular ROS in Raw 264.7 macrophages. (J) Quantification of the relative contents of ROS by using 2′,7′-dichlorodihydrofluorescein diacetate (DCFH-DA) probe in Raw 264.7 macrophages by flow cytometry. Data are expressed as means ± SEM (*n* = 3; ns = no significance).

The internalization profile of MLNs by Raw 264.7 macrophages was similar to that of CT-26 cells, with the strongest green fluorescent intensity in FP127@C6-MLN-treated cells (Fig. 4A). MLNs were internalized into Raw 264.7 macrophages in a time-dependent manner (Fig. S6). It is known that MLLs possess abundant end galactose moieties [30], and these monosaccharide tails are ligands for the galactose lectins, which are massively expressed on the surface of activated macrophages [31]. Accordingly, we presumed that MLNs could naturally target macrophages. To verify this, Raw 264.7 macrophages were incubated in the medium containing free galactose and FP127@C6-MLNs. It was found that the presence of free galactose led to a remarkable decrease in the internalization percentages of FP127@C6-MLNs by macrophages. For instance, 4 h after coincubation, the cellular uptake percentage of the FP127@C6-MLN-treated macrophages was 4.1-fold higher than that of the macrophages with the treatment of FP127@C6-MLNs in the presence of free galactose (400 μg/ml), verifying that MLNs were internalized by macrophages via galactose receptor-mediated endocytosis (Fig. 4B). This observation was consistent with the reduced MFI values of FP127@C6-MLN-treated cells in the presence of free galactose (Fig. S7).

Based on the surface markers, macrophages can be divided into proinflammatory M1 phenotype and anti-inflammatory M2 phenotype [32]. M1-type macrophages preferentially secrete proinflammatory cytokines (e.g., tumor necrosis factor-*α* [TNF-*α*]), while M2-type macrophages tend to produce anti-inflammatory factors (e.g., interleukin-10 [IL-10]) [33]. It was reported that the conversion of M1-type macrophages into M2-type macrophages by certain stimulating factors was beneficial in alleviating inflammatory responses [34]. Accordingly, we investigated the influence of MLNs on the phenotypic polarization of macrophages. As presented in Fig. 4C and D, the control macrophages only receiving the treatment of lipopolysaccharide (LPS) showed the vigorous red fluorescence intensity of M1-type markers (inducible nitric oxide synthase [iNOS]). In contrast, the lowest amount of iNOS was observed in the FP127@RN-MLN-treated macrophages. We further found that the expression levels of M2-type markers (macrophage mannose receptor, CD206) appeared to have an opposite tendency to iNOS, with the weakest green fluorescence intensity in control cells and the strongest green fluorescence intensity in FP127@RN-MLN-treated cells (Fig. 4C and E). All these findings demonstrate that FP127@RN-MLNs have the most vital capacity to promote the transformation of macrophages into M2 phenotype, which is expected to be beneficial to the treatment of inflammatory diseases.

Activated macrophages in the colitis tissues are reported to secrete large amounts of proinflammatory cytokines, further exacerbating the inflammatory responses [35]. Thus, the secretion profiles of the typical inflammatory factors from various MLN-treated macrophages were studied using their corresponding enzyme-linked immunosorbent assay (ELISA) kits. Figure 4F indicated that the negative-control macrophages secreted the least amount of the typical proinflammatory cytokine (TNF-*α*), while the positive-control group (LPS treatment) showed the most considerable TNF-*α* amount. Importantly, MLN treatment greatly decreased the secreted amounts of TNF-*α*, and the FP127@RN-MLN-treated group yielded the lowest quantity of TNF-*α* among all the MLN-treated groups. We further found that the expression levels of the specific anti-inflammatory factor (IL-10) exhibited an opposite trend when compared to the TNF-*α* results (Fig. 4G). ROS is a critical indicator of the inflammatory status, which can cause oxidative damage to the normal tissues due to the lipid peroxidation, protein oxidation, and DNA damage [36]. Inspired by the strong antioxidant activity of MLNs in the acellular environment, we further determined their intracellular antioxidant activity. It was observed that LPS treatment greatly increased the green fluorescence signals within cells, while the FP127@RN-MLN-treated cells exhibited the weakest fluorescence intensity (Fig. 4H and I). Moreover, we quantified the intracellular ROS levels in various cell groups. The treatment of FP127@RN-MLNs attained the lowest levels of intracellular ROS among all the LPS-involved groups (Fig. 4J), consistent with the results in Fig. 4I.

These above observations demonstrate that FP127@RN-MLNs exhibit the strongest capacity to propolarize macrophages to the anti-inflammatory M2 phenotype, down-regulate proinflammatory cytokines, up-regulate anti-inflammatory factors, and relieve intracellular oxidative stress among all MLNs. They might be attributable to their preferential cell internalization by macrophages through the mediation of fluorinated P127 and galactose, constant intracellular release of anti-inflammatory drugs (resveratrol), and inherent antioxidant activity of resveratrol.

### FP127 improves colonic mucus penetration and mucosa accumulation of MLNs

**Fig. 5.**
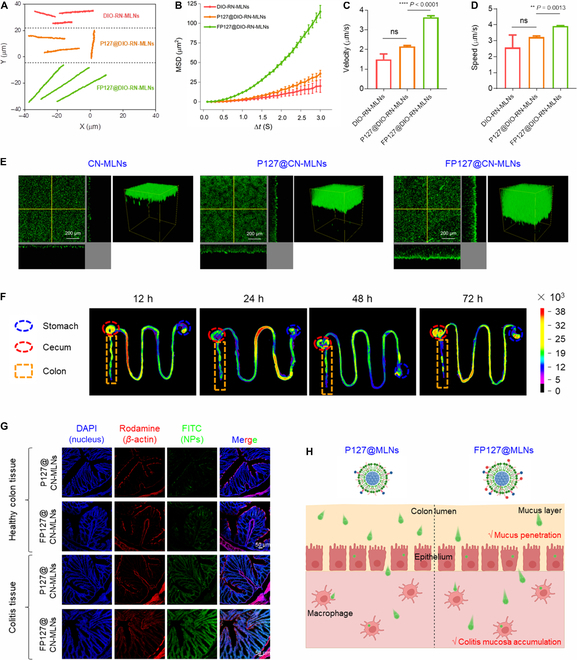
Mucus penetration and in vivo distribution profiles of various MLNs after oral administration. (A) Motion trajectories, (B) MSD values, (C) velocities, and (D) speeds of DIO-RN-MLNs, P127@DIO-RN-MLNs, and FP127@DIO-RN-MLNs in the mucus simulated hydrogels. Data are expressed as means ± SEM (*n* = 3; ns = no significance). (E) 3D fluorescence images of CN-MLNs, P127@CN-MLNs, and FP127@CN-MLNs in the mucus-simulated hydrogels. Scale bar = 200 μm. (F) Ex vivo fluorescence imaging of the GIT from mice receiving oral administration of hydrogel-embedding FP127@DIO-RN-MLNs for 12, 24, 48, and 72 h, respectively. (G) Fluorescence images of the colon sections from mice receiving oral administration of hydrogel-embedding P127@CN-MLNs and FP127@CN-MLNs. Scale bar = 50 μm. (H) Schematic diagram of mucus penetration and mucosa accumulation profiles of P127@MLNs and FP127@MLNs.

The mucus layer separates colonic epithelia from microbiota, which is a functional barrier for both pathogenic microbiota and oral nanomedicines [37]. Thus, we evaluated the mucus-penetrating capacity of MLNs in the simulated mucus. Fig. 5A implied that the movement of RN-MLNs loading with DIO (DIO-RN-MLNs) was impeded in the simulated mucus, which might be attributed to their increased particle sizes in the colonic mucus and their physicochemical interactions with mucus contents. Moreover, the introduction of P127 to the surface of MLNs enhanced their locomotory activities, and this result is consistent with our previous studies [38,39]. Interestingly, the FP127@DIO-RN-MLNs showed the strongest locomotory activity among various MLNs. The mean-square displacement (MSD) has been considered an important factor to reflect the movement ability of NPs [40], and the MSDs of various MLNs were thus assessed. After coincubation of MLNs in the simulated mucus for 3 s, the MSD value of FP127@DIO-RN-MLNs was 5.7- and 3.2-fold higher than those of DIO-RN-MLNs and P127@DIO-RN-MLNs, respectively (Fig. 5B). We further found that FP127@DIO-RN-MLNs presented the highest velocities and speeds among all MLNs (Fig. 5C and D). Three-dimensional fluorescence images illustrated that FP127@MLNs loading with curcumin nanocrystals (FP127@CN-MLNs) achieved the longest permeation distance (Fig. 5E). The improved motion performance of MLNs in the simulated mucus might arise due to their poly(ethylene oxide end chains and fluoroalkanes in FP127, which attenuate the molecular interactions between MLNs and mucoglycoproteins.

Oral nanomedicines are confronted with the harsh intestinal environments, including strong acidic solution, digestive enzymes, and food debris [41]. To protect MLNs during their passage through the GIT, we embedded them into a facilely fabricated hydrogel composed of chitosan and alginate, which were used by our group for oral delivery of various nanoplatforms [42,43]. Energy storage modulus (*G*′) is related to the cross-link density and stiffness of the hydrogel network [44]. Figure S8A revealed that the *G*′ value of chitosan/alginate hydrogel in the presence of Ca^2+^ was observably higher than that of the hydrogel in the absence of Ca^2+^. This observation might be attributed to the fact that the introduction of Ca^2+^ to the chitosan/alginate hydrogel increases its cross-link density. Therefore, this hydrogel can resist the harsh gastrointestinal environment and prevent the premature release of the loaded drugs in the upper GIT. As shown in Fig. S8B, few resveratrol (23.1%) was released from the FP127@CN-MLN-embedded hydrogel in simulated gastric fluid (pH 2.0) and simulated intestinal fluid (pH 7.4) after incubation for 10 h. The percentage of the released resveratrol (54.7%) increased dramatically in simulated colonic fluid (pH 6.8), suggesting that this chitosan/alginate hydrogel could controlled release the encapsulated agents to the colon. To evaluate the in vivo distribution of MLNs, a near-infrared probe (DIO) was encapsulated into MLNs. Subsequently, mice with UC were administered with hydrogel-embedding FP127@DIO-RN-MLNs via the oral route, and MLNs were visualized using an in vivo imaging system. It was observed that at 12 h, the colon tissue showed the most vigorous fluorescence intensity that diminished over time (Fig. 5F and Fig. S8C). In addition, the fluorescence intensities of the 5 principal organs (heart, liver, spleen, lung, and kidney) exhibited a similar decrease trend as those of the colons (Fig. S9), which were much weaker compared with those of the colons at each time point.

To examine the colitis mucosa-accumulating capacity of MLNs, the healthy mice and mice with UC were orally administered with hydrogel-embedding P127@CN-MLNs and FP127@CN-MLNs. Twelve hours after oral administration, the colon tissues were obtained, sectioned, and stained with 4′, 6-diamidino-2-phenylindole and rhodamine-labeled phalloidin. As revealed in Fig. 5G, the healthy colon tissues showed faint green fluorescence signals, whereas the colitis tissues exhibited abundant green signals, suggesting that MLNs preferentially accumulated in the colitis mucosa. These results might be due to the damaged colonic epithelial barriers, allowing for efficient infiltration of MLNs into the mucosa. Notably, the FP127@CN-MLN-treated mice showed noticeably more green fluorescence signals in their colitis mucosa than those treated with P127@CN-MLNs, which could be attributed to the stronger mucus penetration capacity of FP127@CN-MLNs compared with P127@CN-MLNs (Fig. 5H).

### FP127 improves in vivo retardation effect of MLNs against acute UC

**Fig. 6.**
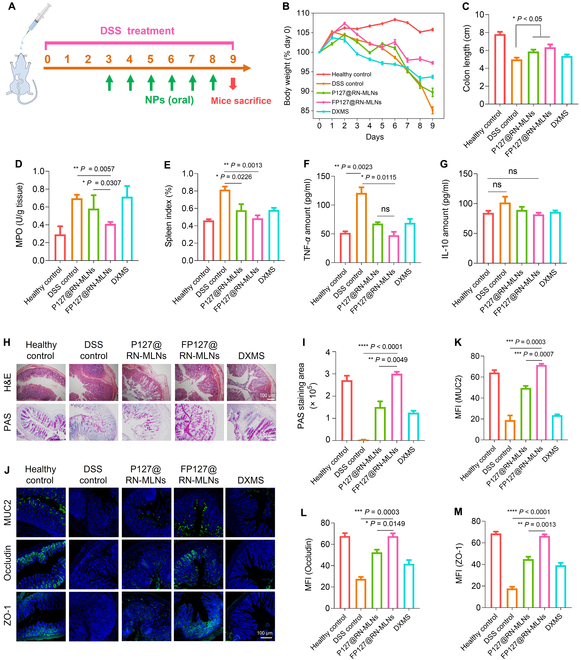
In vivo retardation effects of various MLNs on UC. (A) Schematic illustration of the experimental procedures. Mice were given drinking water or DSS solution (3.5%, w/v) for 9 d and orally administered with hydrogel-embedding P127@RN-MLNs, FP127@RN-MLNs, and DXMS every day on days 3 to 8. (B) Body weight variations over the entire experimental period. (C) Colon lengths, (D) MPO activities, and (E) spleen indexes of various treatment groups. Data are expressed as means ± SEM (*n* = 6). (F) TNF-*α* and (G) IL-10 concentrations in the serum from various treatment groups. (H) H&E and PAS staining of colon sections from various treatment groups. Scale bar = 100 μm. (I) Quantification of PAS staining of the colon sections from various treatment groups. (J) Immunofluorescence staining images of mucin (MUC2) and tight junction proteins (Occludin and ZO-1) from various treatment groups. MUC2, Occludin, and ZO-1 were stained green, and nuclei were stained blue. Scale bar = 100 μm. Relative fluorescence intensities of (K) MUC2, (L) Occludin, and (M) ZO-1 in the colon sections from various treatment groups. Data are expressed as means ± SEM (*n* = 3; ns = no significance).

Making use of the advantageous features of FP127@RN-MLNs, including wound healing, anti-inflammation, antioxidation, and mucus penetration, we further evaluated their retardation effect against UC in vivo. The experimental protocol was shown in Fig. 6A. Mice in the healthy group gained body weight at a steady rate, as opposed to that in the dextran sulfate sodium (DSS) control group, which lost a dramatic amount of body weight by approximately 15.0% (Fig. 6B). As expected, the MLN-treated groups reduced body weight loss, and the FP127@RN-MLN-treated group only lost 2.8% of their body weight, not as much as those in the groups receiving the treatment of P127@RN-MLNs and dexamethasone (DXMS). Colonic length is a typical criterion for determining the severity of UC [45]. Among all groups, the DSS control group had the shortest colon length (5.0 cm), while the FP127@RN-MLN-treated group possessed a significantly longer colon length than the DSS control group (Fig. 6C). Myeloperoxidase (MPO) level is a critical parameter for assessing neutrophil infiltration into the inflamed tissue [46]. The treatment of FP127@RN-MLNs induced the lowest MPO activity among all the treatment groups, including the DXMS-treated group (Fig. 6D). It has been reported that immune cells are activated and proliferated during the development of UC, and preferentially accumulated in the spleen, resulting in the enlargement of spleen [47]. Therefore, the splenic index could be critical in determining UC severity. As illustrated in Fig. 6E, the DSS control group presented a significantly higher spleen index than the other groups, whereas the FP127@RN-MLN-treated group had a spleen index that was equivalent with the healthy control group.

The development of UC is inextricably associated with inflammatory cytokines. TNF-*α* is a critical proinflammatory factor that exacerbates inflammatory reactions and mucosal damage, while IL-10 can down-regulate inflammatory responses and protect colonic epithelial barriers [48]. Figure 6F revealed that compared with the DSS control group, the treatments of P127@RN-MLNs, FP127@RN-MLNs, and DXMS significantly decreased the serum TNF-*α* levels. Notably, the FP127@RN-MLN-treated group possessed the lowest TNF-*α* secretion level among all treatment groups, and its TNF-*α* level was comparable to that in the healthy group. Meanwhile, the healthy control group and the FP127@RN-MLN-treated group showed lower IL-10 concentrations than the DSS control group (Fig. 6G). This phenomenon was inconsistent with the in vitro results (Fig. 4G), which was also observed in our previous reports [7]. As mentioned above, macrophages were incubated with LPS to trigger excessive inflammatory reactions. Cells receiving MLN treatments would secret large quantities of the anti-inflammatory factor (IL-10) to suppress the inflammatory responses. However, in vivo investigations suggested that MLN treatments efficiently relieved UC symptoms, shifting the UC phase from the active status to the recovery status, in which antigen-presenting cells would provide feedback regulation to decrease the secreted amount of IL-10 [49].

Furthermore, hematoxylin-eosin (H&E) staining, periodic acid-Schiff (PAS) staining, and immunofluorescence staining were performed to analyze the histomorphological changes, mucus amounts, and tight junction profiles in the colon tissues (Fig. 6H). It was observed that obvious histological damage were detected in the DSS control group when compared to the healthy control group, including colonic epithelial damage, loss of crypts, reduced goblet cells, and massive accumulation of immune cells. The treatments of P127@RN-MLNs and DXMS obtained much less histological damage, and remarkably, the histological appearance of the colon tissues in the FP127@RN-MLN-treated group was similar to that of the healthy control group, which was reflected by the histological scores (Fig. S10). Previous studies indicated that the mucus layer became thin or even destroyed during UC development [12]. The mucopolysaccharide amount in the DSS control group was the lowest among all the experimental groups. After oral treatment of various MLNs, the mucopolysaccharide amounts clearly increased without significant difference between the FP127@RN-MLN-treated group and the healthy control group. Additionally, the mucopolysaccharide-positive area was dramatically more prominent in the FP127@RN-MLN-treated group than that in the P127@RN-MLN-treated group (Fig. 6I). Mucin (MUC2), a typical mucoprotein, is secreted mainly by goblet cells, which is an important component of the mucus [50]. The MUC2 levels in various groups (Fig. 6J) presented a similar trend as that of mucopolysaccharides detected by PAS staining. The integrity of the colonic epithelium layer was maintained by the tight junction proteins, such as Occludin and zonula occludens protein 1 (ZO-1). Compared with the healthy control group, the DSS control group exhibited much less green fluorescence signals of Occludin and ZO-1; however, the FP127@RN-MLN-treated group showed comparable green signals as the healthy control group (Fig. 6K to M).

The organ indexes (Fig. S11) and H&E-staining results (Fig. S12) of the 5 principal organs revealed the excellent in vivo biosafety of FP127@RN-MLNs via the oral route. Nevertheless, we also noticed that lung tissues from the DSS control group and the DXMS-treated group contained a large number of immune cells, suggesting that the inflammatory severe status in the colon could impact on the inflammatory microenvironment in the lung. Blood samples from each experimental group were analyzed, and no significant difference was found between the healthy control group and the FP127@RN-MLN-treated group (Fig. S13). All these findings demonstrate that oral administration of hydrogel-embedding FP127@RN-MLNs attained excellent therapeutic outcomes and yet negligible systemic adverse effects for UC treatment through increased secretion of mucus, protection of colonic epithelial barriers, and mitigation of colonic inflammation.

### FP127 improves in vivo therapeutic effect of MLNs against acute UC

**Fig. 7.**
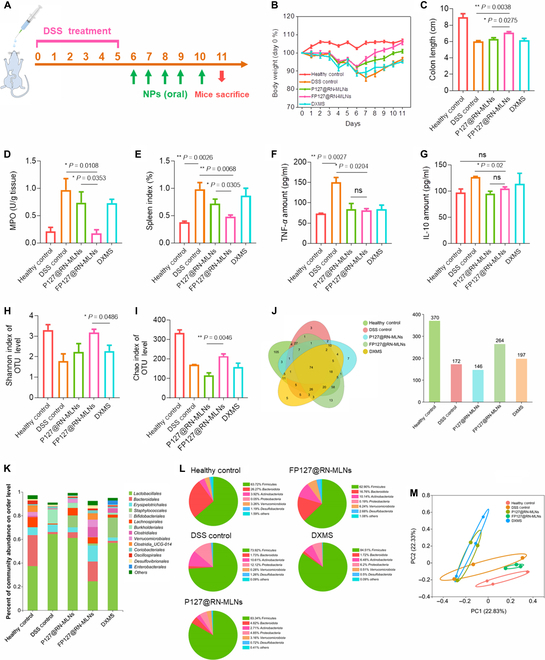
In vivo therapeutic effect of MLNs on acute UC and capacity of MLNs to modulate intestinal microbial compositions. (A) Schematic illustration of the experimental procedures. Mice were given drinking water or DSS solution (3.5%, w/v) for 5 d and orally administered with hydrogel-embedding P127@RN-MLNs, FP127@RN-MLNs, and DXMS every day on days 6 to 10. (B) Body weight variations over the whole experimental period. (C) Colon lengths, (D) MPO activities, and (E) spleen indexes of various treatment groups. Data are expressed as means ± SEM (*n* = 6). Concentrations of (F) TNF-*α* and (G) IL-10 in the serum from various treatment groups. Data are expressed as means ± SEM (*n* = 3; ns = no significance). (H) Shannon and (I) Chao indexes for OUT levels of intestinal microbes from various treatment groups. Data are expressed as means ± SEM (*n* = 3). (J) Venn diagrams of common and unique bacterial species from various treatment groups. (K) The relative abundances of intestinal microbiota from various treatment groups. (L) Microbial compositions of various treatment groups at the phylum level. (M) Principal coordinate analysis of intestinal microflora from various treatment groups. Data are expressed as means ± SEM (*n* = 3).

In practice, patients often take medications after the occurrence of acute UC. To simulate this clinical event, we initially established a mouse model of acute UC by the induction of DSS solution for 5 d, followed by 5 d of oral drug dosages (Fig. 7A). It was observed that all groups involving DSS treatment gradually gained body weight during the period of recovery from UC. Compared with the healthy control group, the DSS control group showed the lowest weight gain efficiency, whereas the FP127@RN-MLN-treated group had the largest body weight gain and reached a comparable body weight of the healthy control group on day 11 (Fig. 7B). Moreover, the colon length in the FP127@RN-MLN-treated group was visibly longer than those in the DSS control group and the P127@RN-MLN-treated group (Fig. 7C). We also found that the treatment of FP127@RN-MLNs remarkably decreased the colonic MPO activity (Fig. 7D), spleen index (Fig. 7E), serum TNF-*α* amounts (Fig. 7F), and serum IL-10 amounts (Fig. 7G), compared with the DSS control group.

H&E staining showed that the DSS control group showed obvious inflammatory signs in the colon tissues. In stark contrast, the FP127@RN-MLN-treated group presented minor epithelial damage and meager accumulated amounts of immune cells, which were similar to the healthy control group (Fig. S14A and B). We further observed that the secreted quantities of mucus contents (mucopolysaccharides and mucoproteins) in the FP127@RN-MLN-treated group were much more than those in other DSS-involved groups (Figs. S14C and S15), including the P127@RN-MLN-treated and DXMS-treated groups. As reported, colitis tissues produce a more amount of sialomucin and a less amount of sulfomucin than the healthy colon tissues, and these 2 types of mucins have critical impacts on the regulation of inflammatory reactions and intestinal microbiota [51,52]. It was observed that the DSS control group showed much higher levels of sialomucin in the colon tissues compared with the healthy control group, and the treatment of FP127@RN-MLNs greatly decreased the secretion of sialomucin. However, sulfomucin presented an opposite variation trend in the colon tissues from various treatment groups. In addition, FP127@RN-MLN treatment greatly increased the expression levels of tight junction proteins (Occludin and ZO-1) compared with other treatment groups (Figs. S16 and S17). It can be found from the literature that the extent of neutrophil infiltration is positively correlated with the severity of UC, and Ly6G and CD11b are considered the hallmarks of neutrophils [53]. Therefore, we investigated the infiltration profiles of neutrophils in the colon tissues using immunofluorescence staining. As revealed in Fig. S18, the DSS control group presented the largest amounts of Ly6G-positive and CD11b-positive cells in the colon tissues. The treatments of MLNs and DXMS greatly reduced the infiltrated levels of neutrophils. Especially, the FP127@RN-MLN-treated group showed the minimum number of infiltrated neutrophils, which was similar to that of the healthy control group. Additionally, FP127@RN-MLN treatment resulted in negligible histopathological damage to the 5 principal organs (Fig. S19), unapparent variations in organ indexes (Fig. S20), and routine blood parameters (Fig. S21), demonstrating their desirable in vivo biosafeties after oral administration.

### MLNs modulate intestinal microbiome homeostasis during the treatment of acute UC

Accumulating evidence has suggested that the pathogenesis of UC is associated with intestinal microbiota, and dysbiosis could trigger metabolic disorders, mucosal damage, and inflammation exacerbation [54,55]. To determine whether MLNs could modulate the intestinal microbiota, we analyzed the compositions of intestinal microbes in the feces at the end of the therapeutic experiments against acute UC. Shannon and Chao indexes implied that the DSS control group showed the lowest microbial community abundance and diversity, which increased obviously in all MLN-treated groups, peaking in the FP127@RN-MLN-treated group (Fig. 7H and I). The Venn plots illustrated that FP127@RN-MLNs efficiently increased the abundance of microbial communities compared with other DSS-involved groups (Fig. 7J). Further data analysis revealed that FP127@RN-MLNs obviously increased the microbial compositions at the order level (Fig. 7K) and the phylum level (Fig. 7L).

The heatmap is used to visualize the similarity in species compositions between the healthy control group and the FP127@RN-MLN-treated group (Fig. S22). The following principal coordinate analysis suggested the different microbial compositions among the experimental groups. It was worth noting that an evident overlap in microbial compositions was found among the DSS control group, the P127@RN-MLN-treated group, and the DXMS-treated group. However, the microbial compositions in the FP127@RN-MLN-treated group were closest to those in the healthy control group, compared with other DSS-involved groups (Fig. 7M). FP127@RN-MLNs were used to achieve a better therapeutic effect against UC by decreasing the relative abundance of harmful species, such as *Parasutterella* and *Oscillibacter*, while increasing the relative abundance of beneficial groups, such as *Clostridium* and *Parabacteroides* (Fig. S23).

### FP127 improves in vivo therapeutic effect of MLNs against chronic UC

**Fig. 8.**
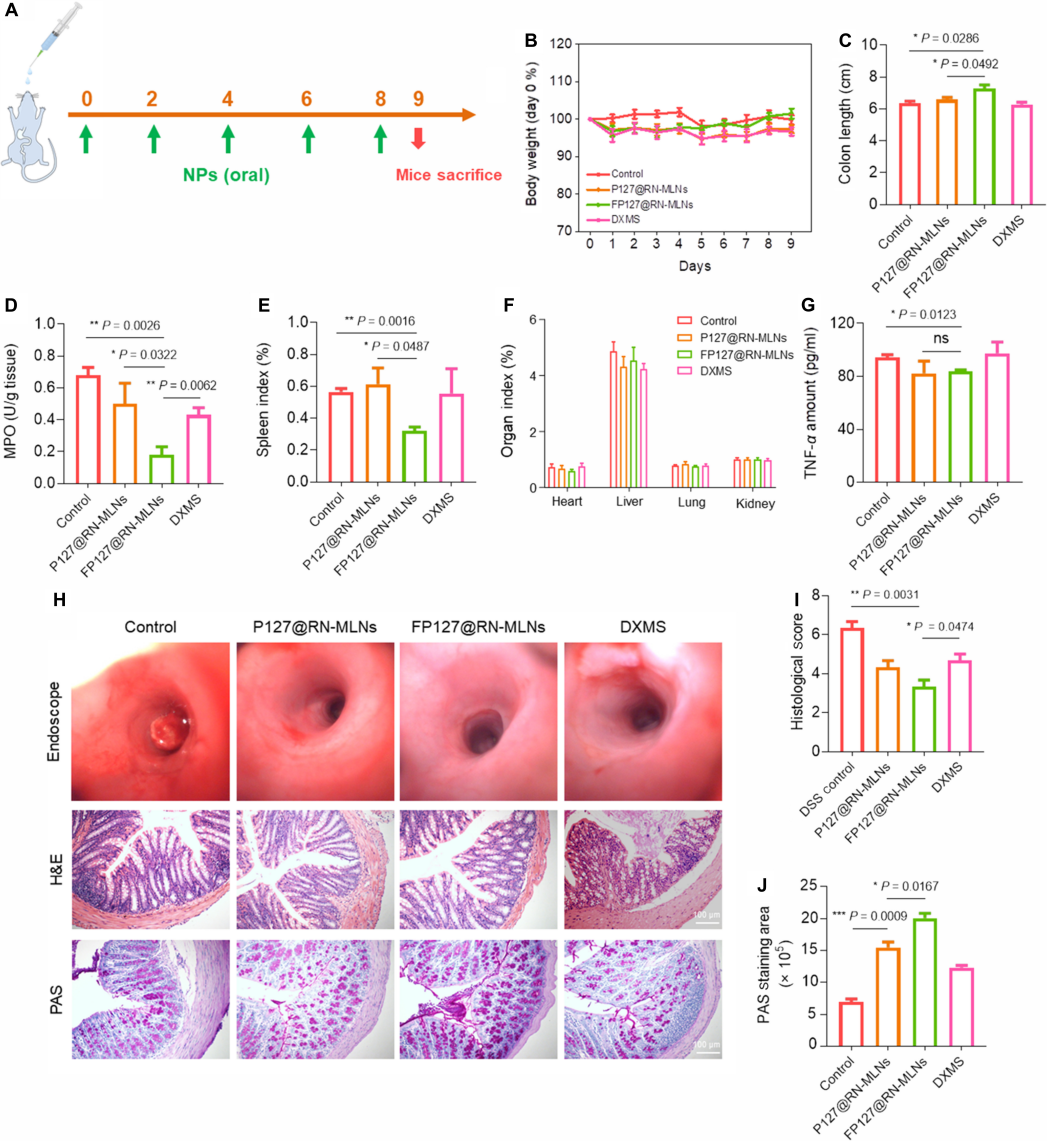
In vivo therapeutic effect of MLNs on chronic UC. (A) Schematic illustration of the experimental procedures. IL-10 knockout mice were orally administered with hydrogel-embedding P127@RN-MLNs, FP127@RN-MLNs, and DXMS every 2 d from days 0 to 8. (B) Body weight variations over the entire experimental period. (C) Colon lengths, (D) MPO activities, and (E) spleen indexes of various treatment groups. Data are expressed as means ± SEM (*n* = 6). (F) Organ indexes of various treatment groups. Data are expressed as means ± SEM (*n* = 6). Concentrations of (G) TNF-*α* in the serum from various treatment groups. Data are expressed as means ± SEM (*n* = 3; ns = no significance). (H) Endoscopic imaging of colon lumens and H&E/PAS-stained images of the colon sections from various treatment groups. Scale bar = 100 μm. (I) Histological scores of the colons from various treatment groups. (J) Quantification of PAS staining of the colon sections from various treatment groups. Data are expressed as means ± SEM (*n* = 3).

Chronic UC is another common type of inflammatory disease in the colon [56]. Accordingly, we investigated the therapeutic outcomes of FP127@RN-MLNs against chronic UC based on IL-10 knockout mice, which could spontaneously develop chronic UC. The treatment protocol was presented in Fig. 8A. It was found that all mouse groups presented stable body weight during the entire treatment period (Fig. 8B). After 8 d of dosing, the FP127@RN-MLN-treated group showed the longest colon length (Fig. 8C), the lowest colonic MPO activity (Fig. 8D), and the smallest spleen index (Fig. 8E) among all groups. In addition, the organ indexes of the heart, liver, lung, and kidney exhibited no significant differences among these 4 groups (Fig. 8F). H&E results indicated that oral administration of hydrogel-embedding P127@RN-MLNs and FP127@RN-MLNs did not cause any damage to the 5 principal organs, whereas the control group and the DXMS-treated group presented a noticeable accumulation of immune cells in the lungs (Fig. S24). The proportions of lymphocytes (Lymph), white blood cells (WBC), and monocytes (Mon) decreased in all treatment groups when compared to the control group, even though no statistically significant difference was found among these groups (Fig. S25). Regarding serum TNF-*α*, the treatment of FP127@RN-MLNs achieved a significantly lower serum TNF-*α* concentration than the control mice (Fig. 8G).

Furthermore, we conducted an endoscopic observation on day 8. It was observed that the control group showed severe intestinal bleeding, and the colon tissues from the P127@RN-MLN- and DXMS-treated groups appeared red and slightly congested. Strikingly, there was no bleeding and erythema at the colonic site in the FP127@RN-MLN-treated group, and its endoscopic appearances resembled that of the normal colon (Fig. 8H), suggesting that FP127@RN-MLNs could efficiently alleviate the symptoms of chronic UC. The protective effect of FP127@RN-MLNs for the colonic epithelial barrier was verified, as evidenced by H&E and PAS results. As seen in Fig. 8H, FP127@RN-MLNs markedly alleviated inflammatory phenomena, such as damage to the epithelial barrier, depletions of goblet cells, infiltrations of immune cells, and disruptions of mucosal crypts. Histological scores were the lowest in the FP127@RN-MLN-treated group compared to the other groups (Fig. 8I). The FP127@RN-MLN-treated group presented more magenta areas (mucus) than the other groups (Fig. 8J).

## Discussion

Over recent decades, there has been a pressing need for exploiting green, effective, and safe drug systems for disease managements [7]. Edible plants have been considered well suited for drug development, as they contain abundant biofunctional ingredients [8]. Resveratrol, a natural polyphenolic compound, is enriched in mulberry, grape, and cranberry, which owns extraordinary benefits for human healthy, including antioxidation, anti-inflammation, immune regulation, and mucosal barrier protection [57]. Notably, the clinical studies reveal that resveratrol supplementation (500 mg/day) greatly mitigates oxidative stress and reduces proinflammatory factors, leading to decreasing the clinical UC activity index and improving the life quality of patients with UC [58]. Unfortunately, the inherent hydrophobicity, high photosensitivity, rapid in vivo metabolism, and lesion-targeting deficiency of resveratrol have restricted its extensive clinical application [59].

The development of nanotechnology has opened new avenues for oral drug delivery, and thus, various forms of NPs, such as liposomes, micelles, and drug nanocrystals, have been exploited recently [5]. Among these NPs, drug nanocrystals are nanoscale drug suspension systems stabilized by trace quantities of surfactants or amphiphilic polymers, which can greatly improve the aqueous drug solubility, drug bioavailability, and drug loading amounts [60]. Therefore, resveratrol was processed into nanocrystals in the present study (Fig. 1A). Considering the 2 clinical UC treatment aims (recovery of damaged colonic epithelial barriers and alleviation of inflammatory responses), colonic epithelia and macrophages have been considered 2 types of target cells [61]. The colonic epithelial cells are made readily accessible, as they are present on the out layer of the colonic mucosa. For macrophages, FP127@RN-MLNs bearing with the end galactoses could be specifically identified by galactose-type lectins on the surface of activated macrophages, resulting in high efficient cellular uptake (Figs. 3A and B and 4A and B).

Once the internalization by the colonic epithelia, FP127@RN-MLNs promoted the migration of these cells to the wounded sites through the activation of cell migration-related signaling pathways associated with *N*-Cadherin and Cyclin D1, resulting in the accelerated recovery of the damaged epithelial barriers (Fig. 3C to G). Meanwhile, the treatment of FP127@RN-MLNs facilitated the transformation of macrophages from the proinflammatory M1 phenotype to the anti-inflammatory M2 type (Fig. 4C to E). We further found that FP127@RN-MLNs significantly decreased the secreted levels of proinflammatory cytokines (TNF-*α*) and increased the levels of anti-inflammatory factors (IL-10) (Fig. 4F and G). Nevertheless, these MLNs maintained the antioxidative activities of resveratrol (Fig. 1H) and reduced the intracellular oxidative stress (Fig. 4H to J). Importantly, the FP127-RN@MLN treatment efficiently relieves the typical symptoms of both acute UC and chronic UC, as evidenced by reduced body weight loss, colon length shortening, decreased MPO values, lowered spleen index, and mitigated inflammatory levels. We also found that mice receiving the treatment of FP127@RN-MLNs had similar colonic histological appearances, goblet cell amounts, mucus contents, and microbial compositions as those displayed by the healthy control group (Figs. S6 to S8). In addition to the satisfactory therapeutic outcomes against acute and chronic UC, it is essential to note that FP127@RN-MLNs also exerted excellent biosafety during the entire treatment period (Figs. S12, S20, and S24).

Collectively, FP127@RN-MLNs act as a noninvasive, safe, and effective therapeutic regimen based on a mouse model of UC. To further confirm their UC treatment outcomes, they have to be tested in clinical practice. Considering that UC is a recurrent inflammatory disease, the preventive effect and long-term safety of FP127@RN-MLNs are necessary to be studied. Nevertheless, it is critical for screening more plants to exploit new lipids with improved biomedical functions, such as enhanced stability in the GIT, intrinsic anti-inflammatory activity, and high drug loading capacity. We believe that the continuing exploitation of plant medicines will facilitate their extensive clinical applications.

## Materials and Methods

### Synthesis of FP127

P127 (160 mg) was dissolved in dioxane (8 ml). Triethylenediamine (1 mg) was used as a catalyst and added to the P127 solution. TFEO (50 μl) was dissolved in dioxane and slowly dropped into the P127 solution. The mixture was kept at 90 °C for 6 h. When cooling down to the room temperature, the mixture containing FP127 was firstly dialyzed against dioxane for 24 h, followed by deionized water for 48 h. Finally, the product was freeze-dried and stored at −20 °C.

### Preparation of RNs and CNs

Resveratrol powders (5 mg) were dissolved in absolute ethanol (2 ml) and quickly added to the polyvinylpyrrolidone aqueous solution (0.05%, w/v). Blue opaline was observed in the solution, and the reaction system was placed on a magnetic stirrer for natural volatilization for 2 h. Similarly, CNs were prepared according to this method.

### Fabrication of various MLNs

Xinjiang medicinal mulberry leaves were washed with tap water to remove impurities, soaked in phosphate buffer saline, placed in the refrigerator at 4 °C overnight, and then milled with a juicer to obtain mulberry leaf juice. Thereafter, the mulberry leaf juice was centrifuged at 12,000 *g* for 2 h to collect the supernatant of mulberry leaf juice, which was then mixed with dichloromethane, methanol, and deionized water, centrifuged at 2,000 *g* for 10 min to collect the lower organic phase, followed by mixing the organic phase with the potassium chloride solution and centrifuging at 2,000 *g* for 10 min to collect the lower organic phase. After washing with water, the lower organic phase obtained through centrifugation was mixed with or without P127 and FP127 (m(P127/FP127):m(BCA total protein) = 6:100), respectively, and then evaporated into a film using a rotary evaporator. Finally, RNs (m(RNs):m(BCA total protein) = 3:160) was added to the film and sonicated in a water bath until the film was completely dissolved, and the prepared NPs were sonicated in a probe at 100-W working power for 2 min. P127@RN-MLNs and FP127@RN-MLNs were sealed and stored in a refrigerator at 4 °C. Similarly, CN-MLNs, P127@CN-MLNs, and FP127@CN-MLNs were prepared similarly for in vivo imaging experiments, and C6-MLNs, P127@C6-MLNs, and FP127@C6-MLNs were prepared for cellular uptake experiments.

### In vitro anti-inflammatory activities of MLNs

To evaluate the in vitro anti-inflammatory effect of various MLNs, Raw 264.7 macrophages were seeded into 12-well plates at a density of 1.0 × 10^5^ cells per well. After overnight incubation, cells were incubated with RN-MLNs, P127@RN-MLNs, and FP127@RN-MLNs (equivalent to 2 μg/ml RNs) for 4 h. Complete medium (500 μl) was supplemented in each well. After 20 h, the suspension was discarded, and cells were stimulated with LPS (1 μg/ml) for 4 h. Subsequently, cell supernatants were collected and centrifuged at 1,000 *g* for 10 min. Finally, the concentrations of proinflammatory factor TNF-*α* and anti-inflammatory factor IL-10 were measured by ELISA. Cells without treatment of LPS and MLNs were used as a negative control, while cells stimulated with LPS but without MLNs were used as a positive control.

### In vivo therapeutic effect of MLNs against UC

Balb/c female mice (6 to 8 wk of age) were randomly divided into 5 groups: the healthy control group, the DSS control group, the P127@RN-MLN-treated group, the FP127@RN-MLN-treated group, and the DXMS-treated group. After giving DSS solution (3.5%, w/v) for 5 d, the DSS solution was replaced with sterile water, while mice were treated with hydrogel-embedding MLNs and DXMS. On day 11, mice were euthanized; colon lengths were measured; and the 5 principal organs and colons were gathered for H&E staining, PAS staining, and immunofluorescent staining (MUC2, Occulidin, ZO-1, CD11b, and Ly6G). Meanwhile, mouse feces were collected for intestinal flora determination. MPO levels were measured by the corresponding kit (Nanjing Jiancheng Bioengineering Institute, Jiangsu, China). Mouse blood was obtained for routine blood analysis (BC-2800 VET, Mindray, Guangdong, China). Inflammatory factors were quantified by the corresponding ELISA kits (Beijing Solarbio Science & Technology Co., Ltd., Beijing, China).

### Statistical analysis

All values are expressed as mean ± standard error of the mean (SEM). Statistical analysis was conducted using Student *t* test or 1-way analysis of variance, unless otherwise noted. Statistical significance was represented by *P < 0.05, **P < 0.01, ***P < 0.001, and ****P < 0.0001.

## Data Availability

All data needed to evaluate the conclusions in the paper are present in the article and/or the Supplementary Materials.

## References

[1] Danese S, Roda G, Peyrin-Biroulet L. Evolving therapeutic goals in ulcerative colitis: Towards disease clearance. Nat Rev Gastroenterol Hepatol. 2020;17(1):1–2. 31520081 10.1038/s41575-019-0211-1

[2] Khan S, Waliullah S, Godfrey V, Khan MAW, Ramachandran RA, Cantarel BL, Behrendt C, Peng L, Hooper LV, Zaki H. Dietary simple sugars alter microbial ecology in the gut and promote colitis in mice. Sci Transl Med. 2020;12(567): Article eaay6218. 33115951 10.1126/scitranslmed.aay6218

[3] Chapman TP, Gomes CF, Louis E, Colombel JF, Satsangi J. De-escalation of immunomodulator and biological therapy in inflammatory bowel disease. Lancet Gastroenterol Hepatol. 2020;5(1):63–79. 31818473 10.1016/S2468-1253(19)30186-4

[4] Troncone E, Monteleone G. The safety of non-biological treatments in ulcerative colitis. Expert Opin Drug Saf. 2017;16(7):779–789. 28608717 10.1080/14740338.2017.1340936

[5] Zu M, Ma Y, Cannup B, Xie D, Jung Y, Zhang J, Yang C, Gao F, Merlin D, Xiao B. Oral delivery of natural active small molecules by polymeric nanoparticles for the treatment of inflammatory bowel diseases. Adv Drug Deliv Rev. 2021;176: Article 113887. 34314785 10.1016/j.addr.2021.113887

[6] Xiao B, Merlin D. Oral colon-specific therapeutic approaches toward treatment of inflammatory bowel disease. Expert Opin Drug Deliv. 2012;9(11):1393–1407. 23036075 10.1517/17425247.2012.730517

[7] Zu M, Xie D, Canup BSB, Chen N, Wang Y, Sun R, Zhang Z, Fu Y, Dai F, Xiao B. 'Green' nanotherapeutics from tea leaves for orally targeted prevention and alleviation of colon diseases. Biomaterials. 2021;279: Article 121178. 34656857 10.1016/j.biomaterials.2021.121178

[8] Chen N, Sun J, Zhu Z, Cribbs AP, Xiao B. Edible plant-derived nanotherapeutics and nanocarriers: Recent progress and future directions. Expert Opin Drug Deliv. 2022;19(4):409–419. 35285349 10.1080/17425247.2022.2053673

[9] Pinedo M, de la Canal L, de Marcos Lousa C. A call for rigor and standardization in plant extracellular vesicle research. J Extracell Vesicles. 2021;10(6): Article e12048. 10.1002/jev2.12048 PMC8077130 33936567

[10] Dad HA, Gu TW, Zhu AQ, Huang LQ, Peng LH. Plant exosome-like nanovesicles: Emerging therapeutics and drug delivery nanoplatforms. Mol Ther. 2021;29(1):13–31. 33278566 10.1016/j.ymthe.2020.11.030 PMC7791080

[11] Zhang M, Xiao B, Wang H, Han MK, Zhang Z, Viennois E, Xu C, Merlin D. Edible ginger-derived nano-lipids loaded with doxorubicin as a novel drug-delivery approach for colon cancer therapy. Mol Ther. 2016;24(10):1783–1796. 27491931 10.1038/mt.2016.159 PMC5112046

[12] Ensign LM, Cone R, Hanes J. Oral drug delivery with polymeric nanoparticles: The gastrointestinal mucus barriers. Adv Drug Deliv Rev. 2012;64(6):557–570. 22212900 10.1016/j.addr.2011.12.009 PMC3322271

[13] Lechanteur A, das Neves J, Sarmento B. The role of mucus in cell-based models used to screen mucosal drug delivery. Adv Drug Deliv Rev. 2018;124:50 –63. 28751201 10.1016/j.addr.2017.07.019

[14] Cao Y, Liu S, Ma Y, Ma L, Zu M, Sun J, Dai F, Duan L, Xiao B. Oral nanomotor-enabled mucus traverse and tumor penetration for targeted chemo-sono-immunotherapy against colon cancer. Small. 2022;18(42): Article e2203466. 36117129 10.1002/smll.202203466

[15] Khutoryanskiy VV. Beyond PEGylation: Alternative surface-modification of nanoparticles with mucus-inert biomaterials. Adv Drug Deliv Rev. 2018;124:140–149. 28736302 10.1016/j.addr.2017.07.015

[16] Pelaz B, del Pino P, Maffre P, Hartmann R, Gallego M, Rivera-Fernandez S, de la Fuente JM, Nienhaus GU, Parak WJ. Surface functionalization of nanoparticles with polyethylene glycol: Effects on protein adsorption and cellular uptake. ACS Nano. 2015;9(7):6996 –7008. 26079146 10.1021/acsnano.5b01326

[17] Chen Q, Gou S, Ma P, Song H, Zhou X, Huang Y, Kwon Han M, Wan Y, Kang Y, Xiao B. Oral administration of colitis tissue-accumulating porous nanoparticles for ulcerative colitis therapy. Int J Pharm. 2019;557:135–144. 30594685 10.1016/j.ijpharm.2018.12.046

[18] Yang M, Lai SK, Wang YY, Zhong W, Happe C, Zhang M, Fu J, Hanes J. Biodegradable nanoparticles composed entirely of safe materials that rapidly penetrate human mucus. Angew Chem Int Ed Engl. 2011;50(11):2597–2600. 21370345 10.1002/anie.201006849 PMC3100893

[19] Li G, Wang S, Deng D, Xiao Z, Dong Z, Wang Z, Lei Q, Gao S, Huang G, Zhang E, et al. Fluorinated chitosan to enhance transmucosal delivery of sonosensitizer-conjugated catalase for sonodynamic bladder cancer treatment post-intravesical instillation. ACS Nano. 2020;14(2):1586–1599. 32011860 10.1021/acsnano.9b06689

[20] Ge C, Yang J, Duan S, Liu Y, Meng F, Yin L. Fluorinated α-helical polypeptides synchronize mucus permeation and cell penetration toward highly efficient pulmonary siRNA delivery against acute lung injury. Nano Lett. 2020;20(3):1738–1746. 32039603 10.1021/acs.nanolett.9b04957

[21] Liu Y, You Y, Lu J, Chen X, Yang Z. Recent advances in synthesis, bioactivity, and pharmacokinetics of pterostilbene, an important analog of resveratrol. Molecules. 2020;25(21):5166. 33171952 10.3390/molecules25215166 PMC7664215

[22] Yu W, Fu YC, Wang W. Cellular and molecular effects of resveratrol in health and disease. J Cell Biochem. 2012;113(3):752–759. 22065601 10.1002/jcb.23431

[23] Cai TT, Ye XL, Li RR, Chen H, Wang YY, Yong HJ, Pan ML, Lu W, Tang Y, Miao H, et al. Resveratrol modulates the gut microbiota and inflammation to protect against diabetic nephropathy in mice. Front Pharmacol. 2020;11:1249. 32973502 10.3389/fphar.2020.01249 PMC7466761

[24] Qiu H, Gong H, Bao Y, Jiang H, Tong W. Reactive oxygen species-scavenging hollow MnO_2_ nanozymes as carriers to deliver budesonide for synergistic inflammatory bowel disease therapy. Biomater Sci. 2022;10(2):457–466. 34882157 10.1039/d1bm01525g

[25] Miao Z, Jiang S, Ding M, Sun S, Ma Y, Younis MR, He G, Wang J, Lin J, Cao Z, et al. Ultrasmall rhodium nanozyme with RONS scavenging and photothermal activities for anti-inflammation and antitumor theranostics of colon diseases. Nano Lett. 2020;20(5):3079–3089. 32348149 10.1021/acs.nanolett.9b05035

[26] Jantan I, Ahmad W, Bukhari SN. Plant-derived immunomodulators: An insight on their preclinical evaluation and clinical trials. Front Plant Sci. 2015;6:655. 26379683 10.3389/fpls.2015.00655 PMC4548092

[27] Araki Y, Mukaisyo K, Sugihara H, Fujiyama Y, Hattori T. Increased apoptosis and decreased proliferation of colonic epithelium in dextran sulfate sodium-induced colitis in mice. Oncol Rep. 2010;24(4):869–874. 20811666 10.3892/or.2010.869

[28] Li Y, Yang X, Yuan JN, Lin R, Tian YY, Li YX, Zhang Y, Wang XF, Xie YH, Wang SW, et al. Ilex rotunda thunb protects against dextran sulfate sodium-induced ulcerative colitis in mice by restoring the intestinal mucosal barrier and modulating the oncostatin M/Oncostatin M receptor pathway. Front Pharmacol. 2022;13: Article 819826. 35645824 10.3389/fphar.2022.819826 PMC9140055

[29] Ashrafizadeh M, Mirzaei S, Hashemi F, Zarrabi A, Zabolian A, Saleki H, Sharifzadeh SO, Soleymani L, Daneshi S, Hushmandi K, et al. New insight towards development of paclitaxel and docetaxel resistance in cancer cells: EMT as a novel molecular mechanism and therapeutic possibilities. Biomed Pharmacother. 2021;141: Article 111824. 34175815 10.1016/j.biopha.2021.111824

[30] Wang Y, Shao S, Guo C, Zhang S, Li M, Ding K. The homogenous polysaccharide SY01-23 purified from leaf of Morus alba L. has bioactivity on human gut bacteroides ovatus and bacteroides cellulosilyticus. Int J Biol Macromol. 2020;158:698–707. 32387599 10.1016/j.ijbiomac.2020.05.009

[31] Zuo L, Huang Z, Dong L, Xu L, Zhu Y, Zeng K, Zhang C, Chen J, Zhang J. Targeting delivery of anti-TNFalpha oligonucleotide into activated colonic macrophages protects against experimental colitis. Gut. 2010;59(4):470–479. 19951904 10.1136/gut.2009.184556

[32] Fuchs AK, Syrovets T, Haas KA, Loos C, Musyanovych A, Mailander V, Landfester K, Simmet T. Carboxyl- and amino-functionalized polystyrene nanoparticles differentially affect the polarization profile of M1 and M2 macrophage subsets. Biomaterials. 2016;85:78–87. 26854393 10.1016/j.biomaterials.2016.01.064

[33] Wang Y, Smith W, Hao D, He B, Kong L. M1 and M2 macrophage polarization and potentially therapeutic naturally occurring compounds. Int Immunopharmacol. 2019;70:459–466. 30861466 10.1016/j.intimp.2019.02.050

[34] Gan J, Dou Y, Li Y, Wang Z, Wang L, Liu S, Li Q, Yu H, Liu C, Han C, et al. Producing anti-inflammatory macrophages by nanoparticle-triggered clustering of mannose receptors. Biomaterials. 2018;178:95–108. 29920405 10.1016/j.biomaterials.2018.06.015

[35] Sunahori K, Yamamura M, Yamana J, Takasugi K, Kawashima M, Yamamoto H, Chazin WJ, Nakatani Y, Yui S, Makino H. The S100A8/A9 heterodimer amplifies proinflammatory cytokine production by macrophages via activation of nuclear factor kappa B and p38 mitogen-activated protein kinase in rheumatoid arthritis. Arthritis Res Ther. 2006;8(3):R69. 16613612 10.1186/ar1939 PMC1526633

[36] Yu Z, Li Q, Wang J, Yu Y, Wang Y, Zhou Q, Li P. Reactive oxygen species-related nanoparticle toxicity in the biomedical field. Nanoscale Res Lett. 2020;15(1):115. 32436107 10.1186/s11671-020-03344-7 PMC7239959

[37] Cornick S, Kumar M, Moreau F, Gaisano H, Chadee K. VAMP8-mediated MUC2 mucin exocytosis from colonic goblet cells maintains innate intestinal homeostasis. Nat Commun. 2019;10(1):4306. 31541089 10.1038/s41467-019-11811-8 PMC6754373

[38] Zhou X, Liu Y, Huang Y, Ma Y, Lv J, Xiao B. Mucus-penetrating polymeric nanoparticles for oral delivery of curcumin to inflamed colon tissue. J Drug Deliv Sci Technol. 2019;52:157–164.

[39] Huang Y, Canup BSB, Gou S, Chen N, Dai F, Xiao B, Li C. Oral nanotherapeutics with enhanced mucus penetration and ROS-responsive drug release capacities for delivery of curcumin to colitis tissues. J Mater Chem B. 2021;9(6):1604–1615. 33471012 10.1039/d0tb02092c

[40] Ma X, Jannasch A, Albrecht UR, Hahn K, Miguel-Lopez A, Schaffer E, Sanchez S. Enzyme-powered hollow mesoporous janus nanomotors. Nano Lett. 2015;15(10):7043–7050. 26437378 10.1021/acs.nanolett.5b03100

[41] Fuhrmann G, Leroux JC. Improving the stability and activity of oral therapeutic enzymes-recent advances and perspectives. Pharm Res. 2014;31(5):1099–1105. 24185592 10.1007/s11095-013-1233-y

[42] Xiao B, Laroui H, Viennois E, Ayyadurai S, Charania MA, Zhang Y, Zhang Z, Baker MT, Zhang B, Gewirtz AT, et al. Nanoparticles with surface antibody against CD98 and carrying CD98 small interfering RNA reduce colitis in mice. Gastroenterology. 2014;146(5):1289–300.e1–19. 24503126 10.1053/j.gastro.2014.01.056 PMC3992175

[43] Xiao B, Viennois E, Chen Q, Wang L, Han MK, Zhang Y, Zhang Z, Kang Y, Wan Y, Merlin D. Silencing of intestinal glycoprotein CD98 by orally targeted nanoparticles enhances chemosensitization of colon cancer. ACS Nano. 2018;12(6):5253–5265. 29860836 10.1021/acsnano.7b08499

[44] Kim J, Zhang G, Shi M, Suo Z. Fracture, fatigue, and friction of polymers in which entanglements greatly outnumber cross-links. Science. 2021;374(6564):212–216. 34618571 10.1126/science.abg6320

[45] Jeon YD, Bang KS, Shin MK, Lee JH, Chang YN, Jin JS. Regulatory effects of glycyrrhizae radix extract on DSS-induced ulcerative colitis. BMC Complement Altern Med. 2016;16(1):459. 27846836 10.1186/s12906-016-1390-8 PMC5111347

[46] Chami B, Martin NJJ, Dennis JM, Witting PK. Myeloperoxidase in the inflamed colon: A novel target for treating inflammatory bowel disease. Arch Biochem Biophys. 2018;645:61–71. 29548776 10.1016/j.abb.2018.03.012

[47] Nelson DA, Petty CC, Bost KL. Infection with murine gammaherpesvirus 68 exacerbates inflammatory bowel disease in IL-10-deficient mice. Inflamm Res. 2009;58(12):881–889. 19544045 10.1007/s00011-009-0059-x

[48] Xu Z, Zhang X, Wang W, Zhang D, Ma Y, Zhang D, Chen M. Fructus mume (Wu Mei) attenuates acetic acid-induced ulcerative colitis by regulating inflammatory cytokine, reactive oxygen species, and neuropeptide levels in model rats. J Med Food. 2022;25(4):389–401. 35438553 10.1089/jmf.2021.K.0155

[49] Fonseca-Camarillo G, Furuzawa-Carballeda J, Llorente L, Yamamoto-Furusho JK. IL-10— and IL-20—Expressing epithelial and inflammatory cells are increased in patients with ulcerative colitis. J Clin Immunol. 2013;33(3):640–648. 23207823 10.1007/s10875-012-9843-4

[50] Van der Sluis M, De Koning BA, De Bruijn AC, Velcich A, Meijerink JP, Van Goudoever JB, Buller HA, Dekker J, Van Seuningen I, Renes IB, et al. MUC2-deficient mice spontaneously develop colitis, indicating that MUC2 is critical for colonic protection. Gastroenterology. 2006;131(1):117–129. 16831596 10.1053/j.gastro.2006.04.020

[51] Deplancke B, Gaskins HR. Microbial modulation of innate defense: Goblet cells and the intestinal mucus layer. Am J Clin Nutr. 2001;73(6):1131S–1141S. 11393191 10.1093/ajcn/73.6.1131S

[52] Croix JA, Carbonero F, Nava GM, Russell M, Greenberg E, Gaskins HR. On the relationship between sialomucin and sulfomucin expression and hydrogenotrophic microbes in the human colonic mucosa. PLOS ONE. 2011;6(9): e24447. 21931721 10.1371/journal.pone.0024447 PMC3170330

[53] Zhang Q, Zhang C, Chang F, Liang K, Yin X, Li X, Zhao K, Niu Q, Tian Z. Wip 1 inhibits intestinal inflammation in inflammatory bowel disease. Cell Immunol. 2016;310:63–70. 27687530 10.1016/j.cellimm.2016.07.012

[54] Morgan XC, Tickle TL, Sokol H, Gevers D, Devaney KL, Ward DV, Reyes JA, Shah SA, LeLeiko N, Snapper SB, et al. Dysfunction of the intestinal microbiome in inflammatory bowel disease and treatment. Genome Biol. 2012;13(9):R79. 23013615 10.1186/gb-2012-13-9-r79 PMC3506950

[55] Lee Y, Sugihara K, Gillilland MG III, Jon S, Kamada N, Moon JJ. Hyaluronic acid-bilirubin nanomedicine for targeted modulation of dysregulated intestinal barrier, microbiome and immune responses in colitis. Nat Mater. 2020;19(1):118–126. 31427744 10.1038/s41563-019-0462-9 PMC6923573

[56] Tolstanova G, Khomenko T, Deng X, Szabo S, Sandor Z. New molecular mechanisms of the unexpectedly complex role of VEGF in ulcerative colitis. Biochem Biophys Res Commun. 2010;399(4):613–616. 20682292 10.1016/j.bbrc.2010.07.124

[57] Singh AP, Singh R, Verma SS, Rai V, Kaschula CH, Maiti P, Gupta SC. Health benefits of resveratrol: Evidence from clinical studies. Med Res Rev. 2019;39(5):1851–1891. 30741437 10.1002/med.21565

[58] Samsami-Kor M, Daryani NE, Asl PR, Hekmatdoost A. Anti-inflammatory effects of resveratrol in patients with ulcerative colitis: A randomized, double-blind, placebo-controlled pilot study. Arch Med Res. 2015;46(4):280–285. 26002728 10.1016/j.arcmed.2015.05.005

[59] Ma Y, Duan L, Sun J, Gou S, Chen F, Liang Y, Dai F, Xiao B. Oral nanotherapeutics based on Antheraea pernyi silk fibroin for synergistic treatment of ulcerative colitis. Biomaterials. 2022;282: 121410. 35202934 10.1016/j.biomaterials.2022.121410

[60] Chan HK, Kwok PC. Production methods for nanodrug particles using the bottom-up approach. Adv Drug Deliv Rev. 2011;63(6):406–416. 21457742 10.1016/j.addr.2011.03.011

[61] Xiao B, Zhang Z, Viennois E, Kang Y, Zhang M, Han MK, Chen J, Merlin D. Combination therapy for ulcerative colitis: Orally targeted nanoparticles prevent mucosal damage and relieve inflammation. Theranostics. 2016;6(12):2250–2266. 27924161 10.7150/thno.15710 PMC5135446

