# Supplementary material for: Mulberry Biomass-Derived Nanomedicines Mitigate Colitis through Improved Inflamed Mucosa Accumulation and Intestinal Microenvironment Modulation
Source: Research (Wash D C). 2023 Jul 7;6:0188. doi: 10.34133/research.0188 (PMC10328391; doi:10.34133/research.0188)
Supplement: Supplementary 1 — Figs. S1 to S25 [file research.0188.f1.docx]

**Supplementary Materials for**

**Mulberry Biomass-Derived Nanomedicines Mitigate Colitis through Improved Inflamed Mucosa Accumulation and Intestinal Microenvironment Modulation**

Wenjing Yang, Ya Ma, Haiting Xu, Zhenhua Zhu, Jiaxue Wu, Cheng Xu, Wei Sun^1^, Erhu Zhao^1^, Min Wang^*^, Rui L. Reis, Subhas C. Kundu, Xiaoxiao Shi^*^, and Bo Xiao^*^

**1. Experimental Section**

*1.1. Materials*

Mulberry leaves were obtained from Xinjiang Hetian Institute of Sericulture Science, Xinjiang, China. Resveratrol, C6, TFEO, chitosan, sodium alginate, MTT, dimethyl sulfoxide (DMSO), and Triton X-100 were obtained from Aladdin (Shanghai, China). Curcumin was obtained from Dalian Meilun Biology Technology Co., Ltd. (Dalian, China). LPS was purchased from Sigma-Aldrich (St. Louis, MO, USA). CD206/MRC1 rabbit monoclonal antibody, iNOS rabbit monoclonal antibody, FITC-labeled goat anti-rabbit IgG (H + L), Cy3-labeled goat anti-rabbit IgG (H + L), DiO, DAPI, galactose, and ABTS and ROS assay kits were obtained from Beyotime Biotechnology Institute (Nanjing, China). *N*-cadherin monoclonal antibody, cyclin D1 monoclonal antibody, and *α*-tubulin monoclonal antibody were purchased from Proteintech Group, Inc. (Wuhan, China). All ELISA kits were provided by Beijing Solarbio Science & Technology Co., Ltd. (Beijing, China). DSS (36-50 kDa) was purchased from MP Biomedicals (Aurora, OH, USA).

*1.2. Physicochemical Properties of Various MLNs*

Hydrodynamic particle sizes and zeta potentials of MLNs were measured by DLS (Malvan, Zetasizer Nanoz, UK), and the average values were obtained after measurement 3 times. A small drop of diluted MLNs was added to a clean silicon wafer, air-dried overnight, and observed by AFM (Seiko Instruments Inc., Chiba, Japan). The ^1^H NMR spectra of P127 and FP127 were detected on a Bruker Avance spectrometer (600 MHz10600, Bruker, German) using deuterated DMSO as a solvent. The suspensions of RNs, RN@MLNs, P127@RN-MLNs, and FP127@RN-MLNs (equivalent to 300 μg RNs) were added to the dialysis bags (MWCO = 8000-14000 Da) that were then sealed at both ends and transferred to centrifuge tubes (pH = 6.8) with a releasing buffer containing 0.1% (w/v) Tween-80. These tubes were placed in a shaker at 120 rpm/min at 37 °C. The releasing buffer was taken from the tubes to measure the released RN amounts using a microplate reader (Perkin Elmer, Boston, MA, USA). The dialysis bags were placed in centrifuge tubes with a fresh releasing buffer.

*1.3. Fabrication of Fluorescent Dye DIO-loaded MLNs*

MLNs were labeled with lipophilic fluorescent dyes by mixing DIO with various MLNs and incubated in a water bath at 37 °C for 30 min, followed by centrifugation at 12,000 *g* for 30 min to remove the supernatant. Precipitation was collected for the subsequent experiments.

*1.4. Stability of MLNs in Colonic Simulation Buffers*

The suspensions of RNs, RN-MLNs, P127@RN-MLNs, and FP127@RN-MLNs were prepared in the colonic simulation solution (pH = 6.8). These suspensions were placed in a shaker at 120 rpm/min at 37 °C. The average hydrodynamic particle sizes and zeta potentials of various MLNs were measured by DLS at different time points.

*1.5. Antioxidant Property of MLNs*

ABTS was oxidized to green ABTS^•+^ under the action of appropriate oxidants. The production of ABTS^•+^ was inhibited in the presence of antioxidants. The total antioxidant capacity of MLNs was detected and calculated by measuring the absorbance of ABTS^•+^ at 414 nm. The peroxidase working solution was added to each well of the 96-well plate, followed by the sample to be tested. A series of Trolox standard solutions at different concentrations were used as standard curve test wells, while distilled water was served as a negative control. After mixing gently, the ABTS working solution was added to each well. After incubation at room temperature for 6 min, the absorbance at 414 nm was determined as A_414_. The total antioxidant capacity of MLNs was calculated using the standard curve equation to determine the mM of Trolox standard that the sample was equivalent to.

*1.6. Cytocompatibility Test of MLNs*

CT-26 cells and Raw 264.7 macrophages were seeded into 96-well plates at a density of 1.0 × 10^4^ cells per well and incubated overnight. RN-MLN, P127@RN-MLN, and FP127@RN-MLN suspensions (containing 0.5, 1, 2, 3, and 4 μg/mL RNs) were added to each well. After 24 h of co-incubation, the supernatants were discarded, and cells were rinsed 3 times with PBS. MTT solutions were added to each well (100 μL MTT per well). After incubating for 4 h, the supernatants were discarded, and 100 μL DMSO was added to each well. The 96-well plates were placed on a shaker (100 rpm/min) for 15 min, and the OD values at 570 nm were measured by a PerkinElmer EnSpire multimode plate reader (Perkin Elmer, Boston, MA, USA). Untreated cells were considered as a negative control, and cells treated with Triton X-100 (0.5%, w/v) were treated as a positive control.

*1.7. Visualization of Intracellular Uptake of MLNs*

CT-26 cells and Raw 264.7 macrophages were inoculated into a 6-well plate with a density of 2.0×10^5^ cells per well. After incubation overnight, cells were co-cultured with C6-MLN, P127@C6-MLN, and FP127@C6-MLN suspensions (equivalent to 1 μg/mL C6) for 1, 2, and 4 h. Afterwards, cells were rinsed 3 times with PBS, fixed in a paraformaldehyde solution (4%, v/v) for 30 min, and stained with Rhodamine-labeled phalloidin (1:200) for F-actin for 1 h. Nuclei were stained with DAPI (1:500) for 5 min. Finally, cells were imaged using CLSM (Olympus, FV-3000, Japan). MFIs of each experimental group were measured by Image J software.

*1.8. Quantification of Cellular Uptake Efficiencies of MLNs*

CT-26 cells and Raw 264.7 macrophages were seeded into 12-well plates at a density of 2.0 × 10^5^ cells per well and incubated overnight. C6-MLN, P127@C6-MLN, FP127@C6-MLN, and FP127@C6-MLN (+ free galactose) suspensions (equivalent to 0.5 μg/mL C6) were incubated with cells for 1, 2, and 4 h, respectively. Subsequently, cells were digested with trypsin, collected by centrifugation at 1,000 *g* for 4 min, re-suspended in PBS, transferred to 1.5 mL EP tubes, and detected by FCM (ACEA NovoCytet, USA).

*1.9. In Vitro ROS Clearance Capacity of MLNs*

Raw 264.7 macropha*g*es were grown in 12-well plates at a density of 1.0 × 10^5^ cells per well and incubated overnight. RN-MLN, P127@RN-MLN, and FP127@RN-MLN suspensions (equivalent to 2 µg/mL RNs) were added to each well and incubated with cells for 4 h. Thereafter, each well was supplemented with 500 µL of complete medium. After 20 h, MLN suspensions were discarded, and cells were stimulated with LPS (1 µg/mL) for 4 h. DCFH-DA active oxygen fluorescent probe was added to each well and incubated at 37 °C for 20 min. After washing with PBS 3 times, the nuclei were stained with DAPI for 20 min, and cells were visualized using a CLSM (Olympus, FV-3000, Japan). MFIs of each experimental group were measured by Image J software.

Raw 264.7 macrophages were inoculated into 12-well plates at a density of 1.0 × 10^5^ cells per well. After incubation overnight, RN-MLN, P127@RN-MLN, and FP127@RN-MLN suspensions (equivalent to 2 µg/mL RNs) were incubated with cells for 4 h. Thereafter, each well was supplemented with 500 µL of the complete medium. After 20 h, drug suspensions were discarded, and cells were stimulated with LPS (1 µg/mL) for 4 h. DCFH-DA active oxygen fluorescent probe was added to each well and incubated at 37 °C for 20 min. After washing with PBS 3 times, cells were digested with trypsin, collected through centrifugation at 1,000 *g* for 4 min, re-suspended in PBS, transferred to 1.5 mL EP tubes, and detected by FCM (ACEA NovoCytet, USA).

*1.10. In Vitro Wound Healing Properties of MLNs*

To investigate the effect of MLNs on wound healing, CT-26 cells were seeded into 6-well plates at a density of 1.0 × 10^6^ cells per well. After incubation overnight, a pipet tip was used to scratch the wells. After washing twice with PBS, RN-MLN, P127@RN-MLN, and FP127@RN-MLN suspensions (equivalent to 2 μg/mL RNs) were incubated with cells. At pre-determined time points (24 and 48 h), images were taken with a microscope (BX63, Olympus Corporation, Japan), and the scratch width was evaluated by Image J software.

*1.11. Western Blot Assay*

To investigate the mechanism of the effect of FP127@RN-MLNs on cell migration, the variations of proteins associated with the migration signaling pathway (*N*-cadherin and cyclin D1) were assayed by Western Blot experiments. Initially, FP127@RN-MLN suspensions were co-incubated with CT-26 cells for 24 h. Cells were digested with trypsin, collected through centrifugation at 1,000 *g* for 4 min, and lysed on ice with the RIPA buffer for 1 h. The supernatant was collected by centrifugation to detect the total protein concentration. Proteins in the supernatants were separated by sodium dodecyl sulfate-polyacrylamide gel (SDS-PAGE) electrophoresis and transferred to PVDF membranes, which were sealed with skimmed milk powders (5%, w/v) for 2 h. The corresponding primary antibodies (anti-*N*-cadherin, anti-cyclin D1, and anti-*α*-tubulin) and the secondary antibodies were diluted 1,000 times, respectively. The membranes were incubated with primary antibodies overnight at 4 °C and rinsed with the TBST buffer. After rinsing, the membranes were incubated with secondary antibodies for 2 h and washed with the TBST buffer. Final detection was performed using the Western Blotting detection instruments (Clinx Science, Shanghai, China).

*1.12. In Vitro Phenotypic Polarization Profiles of Macrophages*

Raw 264.7 macrophages were seeded in 12-well plates at a density of 1.5 × 10^5^ cells per well. After 8 h, the cell medium was replaced with LPS solution (500 ng/mL), and cells were further cultured for 16 h. RN-MLN, P127@RN-MLN, and FP127@RN-MLN suspensions (equivalent to 2 μg/mL RNs) were co-cultured with cells for 4 h. Each well was supplemented with 500 μL of the complete medium. After 20 h, cells were rinsed with PBS for 3 times, which was fixed with the paraformaldehyde solution (4%, w/v) for 10 min and soaked in Triton X-100 solution (0.1%, w/v) for 10 min before being sealed with the BSA protein solution (1%, w/v). CD206 rabbit polyclonal antibody and iNOS rabbit polyclonal antibody were diluted 500 times, respectively, and cultured with macrophages at 4 °C for 24 h. The primary antibody was removed before cells were washed 3 times with PBS. The FITC-labeled goat anti-rabbit antibody Ig G (H + L) and the Cy3-labeled goat anti-rabbit antibody Ig G (H + L) were diluted 500 times, respectively, and incubated with cells for 1 h at 37 °C. The nuclei were stained with DAPI. Eventually, cells were imaged with CLSM (Olympus, FV-3000, Japan). The average fluorescence intensity of each experimental group was quantified by Image J software.

*1.13. Motion Performance and Mucus-Penetrating Capacities of MLNs*

DIO-RN-MLNs, P127@DIO-RN-MLNs, and FP127@ DIO-RN-MLNs were dispersed in the colonic simulation solution (pH = 6.8). Subsequently, a drop of each suspension was added to a small circular dish containing simulated mucus medium (hydroxyethyl cellulose, 0.6 %, w/v), and the movement of MLNs was recorded with a fluorescent microscope fitted with a CCD camera (Olympus Corporation, Japan) at a phase difference of approximately 30 frames/s. The trajectory/mean square displacement (MSD), mean velocity and mean speed of MLNs were obtained using Cellsens software.
 CN-MLNs, P127@CN-MLNs, and FP127@CN-MLNs were dispersed in the colonic simulation solution (pH = 6.8). A drop of each suspension was added to a 96-well plate containing a simulated mucus medium (hydroxyethyl cellulose, 0.6%, w/v). Three-dimensional fluorescence imaging was performed using CLSM (Olympus, FV-3000, Japan).

*1.14. Ex Vivo Imaging of MLNs*

IL-10 knockout mice with colitis symptoms were orally administered with chitosan/sodium alginate (w/w = 1:1) hydrogel-embedding FP127@DIO-RN-MLNs (equivalent to 3 mg/kg RNs). To prevent NP degradation in the upper GIT, they were encapsulated in the chitosan-alginate hydrogel (3:7 w/w) (<https://protocolexchange.researchsquare.com/article/nprot-588/v1>), which has been commonly used in our group [1-5]. The rheological properties of chitosan/sodium alginate hydrogels with/without calcium ions were determined using a rheometer (MCR 302, Anton Paar, Austria). In a parallel plate configuration, the gels were examined at 37°C at a gap distance of 1 mm. Hydrogels (2 mL) were added to the bottom plate. The strain was set to 1%, and variations in energy storage modulus and loss modulus were recorded over a range of angular frequencies from 0.628 to 6.28 rad/s. The release behaviors of drugs from FP127@RN-MLN-embedded hydrogel were examined in gradually changing buffers: simulated gastric fluid (pH 2.0), simulated intestinal fluid (pH 7.4), and simulated colonic fluid (pH 6.8). FP127@RN-MLN-embedded hydrogel were added to the dialysis bags (MWCO = 8000-14000 Da) that were then sealed at both ends and transferred to centrifuge tubes (pH 2.0, 7.4, and 6.8) with a releasing buffer containing Tween-80 (0.1%, w/v). These tubes were placed in a shaker at 120 rpm/min at 37 °C. The releasing buffer was taken from the tubes to measure the released RN amounts using a microplate reader (Perkin Elmer, Boston, MA, USA). The dialysis bags were placed in centrifuge tubes with a fresh releasing buffer.

Mice were sacrificed at pre-determined time points (12, 24, 48, and 72 h), and the whole GIT and five principal organs (heart, liver, spleen, lung, and kidney) were collected, which were imaged by an *in vivo* imaging system (CELLGENTEK Co., Ltd. Korea). MFIs of the organs were quantified by the system-adapted software.

IL-10 knockout mice with colitis symptoms were orally administered with hydrogel-embedding P127@CN-MLNs or FP127@CN-MLNs (equivalent to 5 mg/kg CNs). Twelve hours after oral administration, mice were sacrificed, and the colon tissues were collected and fixed in the paraformaldehyde solution (4%, v/v). Subsequently, the colon tissues were embedded in an OCT embedding agent at the optimal cutting temperature and cut into 5 µm-thick sections with a freezing microtome. The colon tissue sections were stained with Rhodamine-labeled phalloidin (1:200) for 1 h, followed by DAPI staining for 10 min, and imaged by CLSM (Olympus, FV-3000, Japan).

*1.15. In Vivo Retardation Effect of MLNs against UC*

Balb/c female mice (6–8 weeks of age) were randomly divided into 5 groups: the healthy control, the DSS control, the P127@RN-MLN-treated group, the FP127@RN-MLN-treated group, and the DXMS-treated group. The UC mouse model was established through the drinking DSS aqueous solution (3.5%, w/v) continuously. Hydrogel-embedding MLNs and DXMS were orally administered to mice. During the experiment, mouse body weight and feces were recorded. On day 9, mice were euthanized, and the colon length was measured. Besides, the five principal organs and colons were gathered for H&E staining, PAS staining, and immunofluorescent staining (MUC2, Occulidin, and ZO-1). MPO levels were measured by the corresponding kit (Nanjing Jiancheng Bioengineering Institute, Jiangsu, China). Mouse blood was obtained for routine blood analysis (BC-2800 VET, Mindray, Guangdong, China). Inflammatory factors were quantified by the corresponding ELISA kits (Beijing Solarbio Science & Technology Co., Ltd., Beijing, China).

*1.16. Impact of MLNs on Intestinal Microbiota*

At the end of the treatment experiments on UC mice, mouse feces were collected, and the total DNA from the samples was extracted. The amplification was performed using primer 338F (5′-GTGCCAGCMCMCGCGG-3′) and 806R (5′-CCGTCAATTCMTTTRAGTTT-3′) as templates, followed by sequencing of the gut microbiota by 16S rRNA. The results were analysed based on an interactive platform at Shanghai Meijer Biopharmaceutical Technology Co, Ltd. (Shanghai, China). Primarily, the data were processed for microbial diversities, abundance values and community structures of the samples.

*1.17. In Vivo Therapeutic effect of MLNs on Chronic Colitis*

IL-10 knockout mice that developed colitis symptoms were randomly divided into 4 groups: the control group, the P127@RN-MLN-treated group, the FP127@RN-MLN-treated group, and the DXMS-treated group. Hydrogel-embedding MLNs and DXMS were orally administered to mice. During the experiment, mouse body weight and feces were recorded. On day 9, mice were euthanized, and the colon length was measured. Besides, the five principal organs and colons were gathered for H&E staining, PAS staining, and high iron diamine and alcian blue (HID-Ab) staining. MPO levels were measured by the corresponding kit (Nanjing Jiancheng Bioengineering Institute, Jiangsu, China). Mouse blood was obtained for routine blood analysis (BC-2800 VET, Mindray, Guangdong, China). Inflammatory factors were quantified with the corresponding ELISA kits (Beijing Solarbio Science & Technology Co., Ltd., Beijing, China).


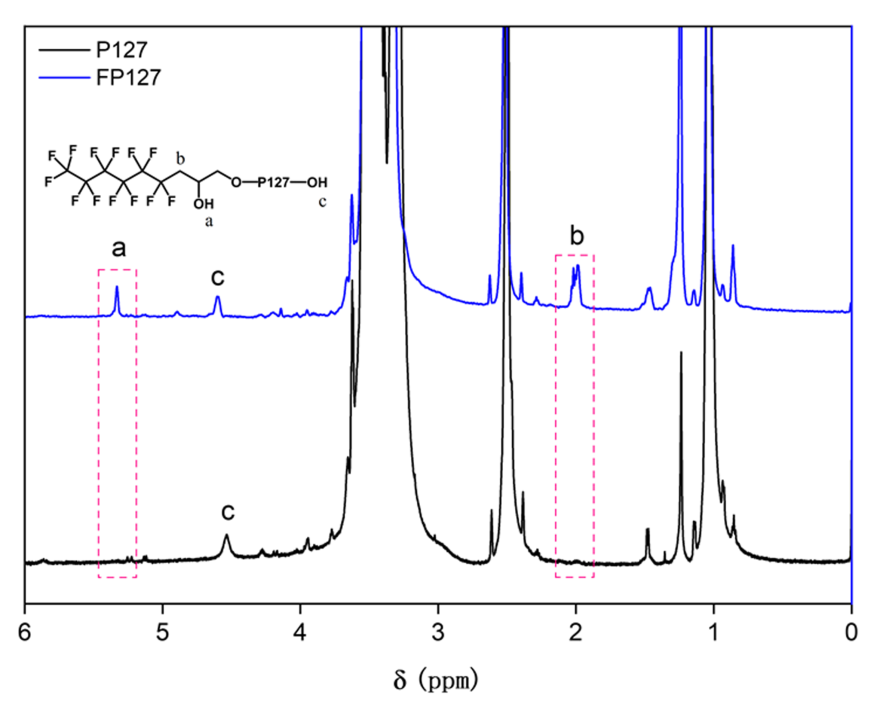


**Fig. S1.** ^1^H NMR spectra of P127 and FP127.


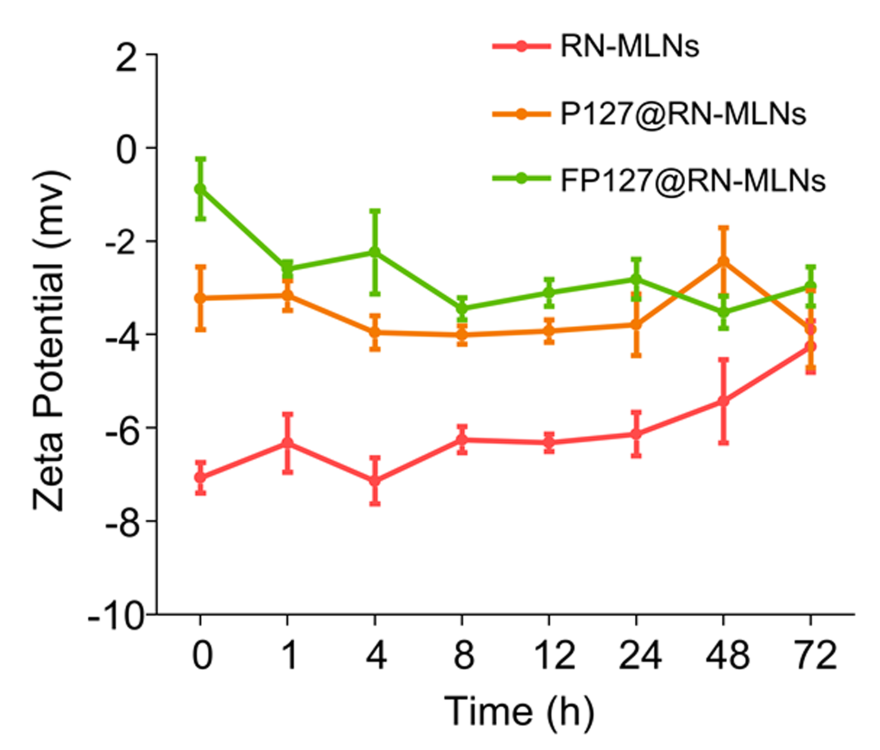


**Fig.S2.** Variations of zeta potentials of RN-MLNs, P127@RN-MLNs, and FP127@RN-MLNs in the colonic simulation solutions (pH = 6.8) for 72 h. Data are expressed as means ± S.E.M. (n = 3).


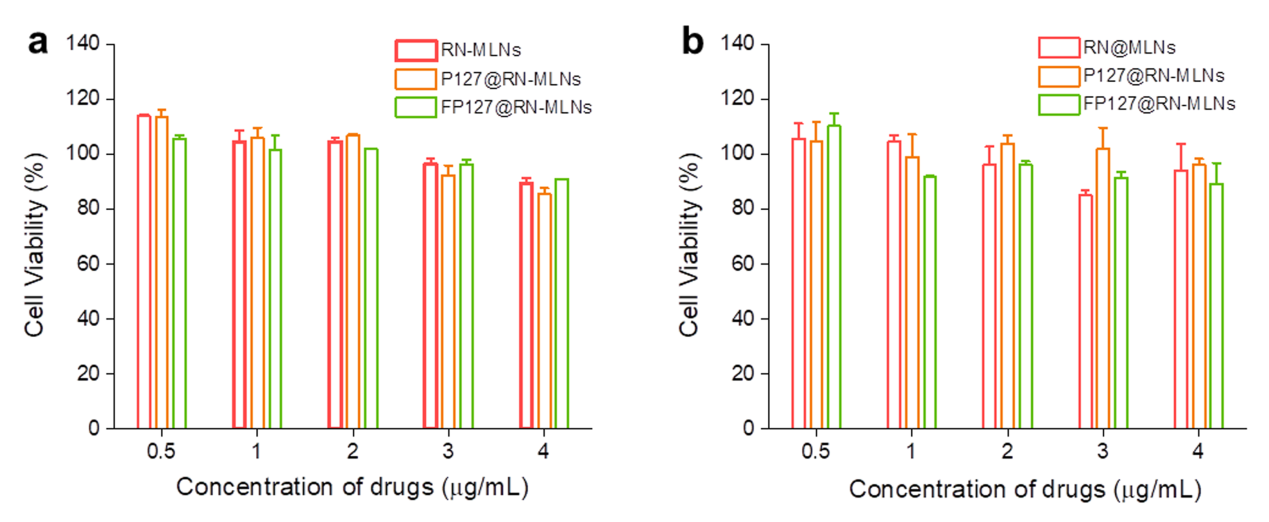


**Fig. S3.** Viabilities of (a) CT-26 cells and (b) Raw 264.7 macrophages with the treatment of various MLNs for 24 h. Untreated cells were served as a negative control, while triton X-100-treated cells were served as a positive control. Data are expressed as means ± S.E.M. (n = 5).


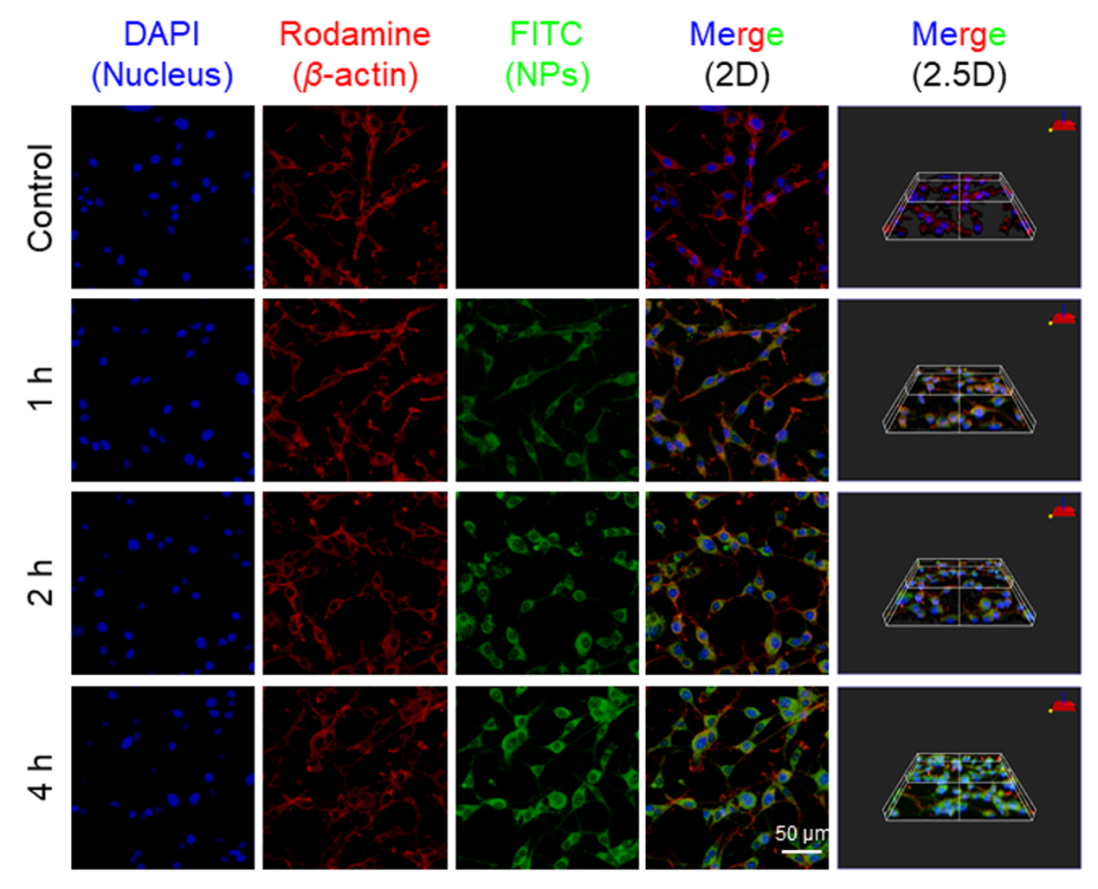


**Fig. S4.** Fluorescence images of *in vitro* uptake profiles of FP127@C6-MLNs by CT-26 cells after co-incubation for 1, 2, and 4 h, respectively. Untreated cells were served as a negative control. Scale bar = 50 μm.


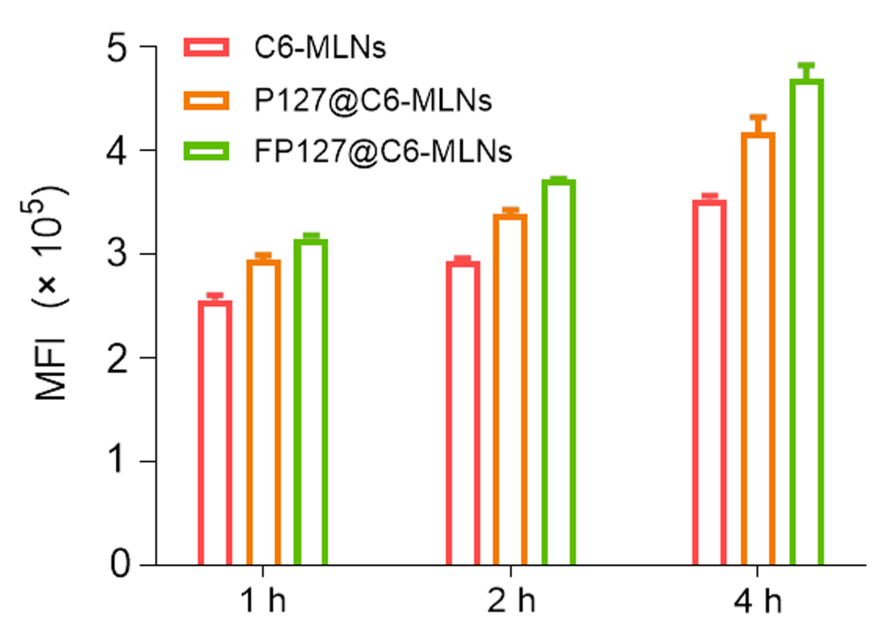


**Fig. S5.** Green fluorescence intensities of CT-26 cells with the treatment of C6-MLNs, P127@C6-MLNs, and FP127@C6-MLNs for 1, 2, and 4 h, respectively. Data are expressed as means ± S.E.M. (n = 3).


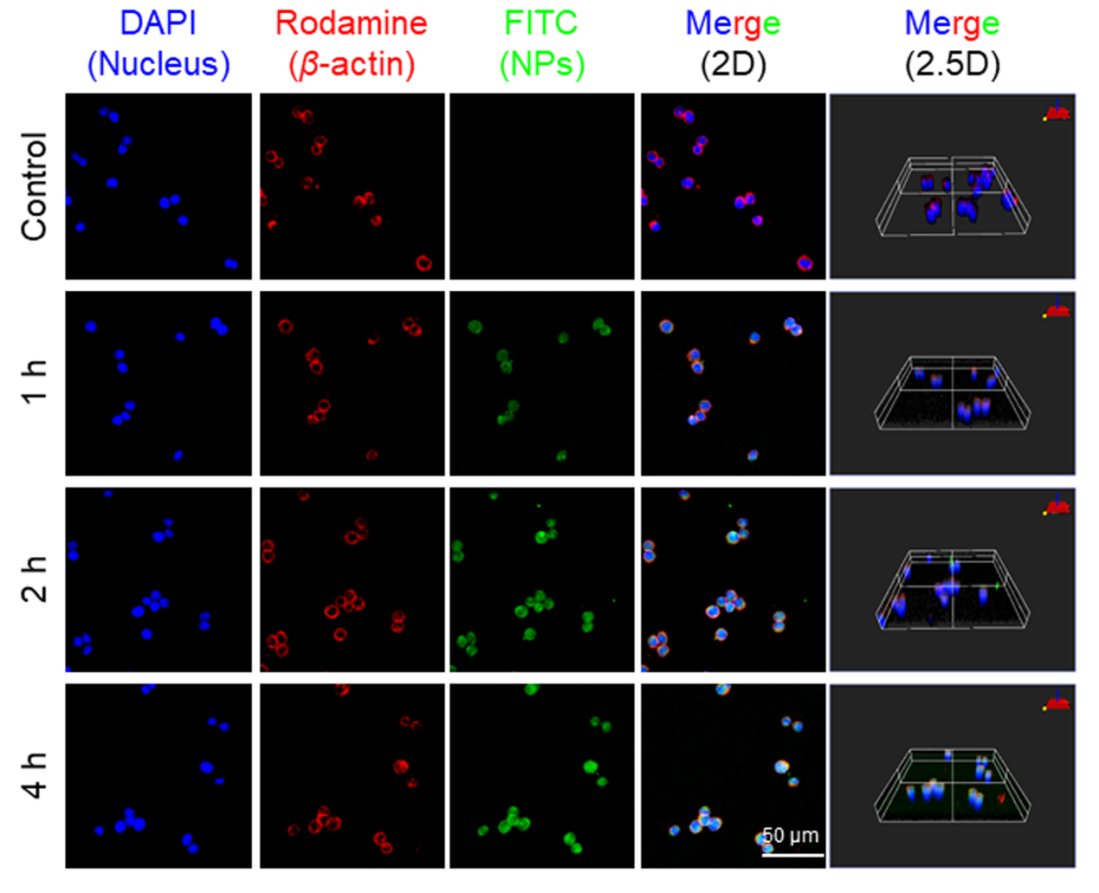


**Fig. S6.** Fluorescence images of *in vitro* uptake profiles of FP127@C6-MLNs by Raw 264.7 macrophages after co-incubation for 1, 2, and 4 h, respectively. Untreated cells were served as a negative control. Scale bar = 50 μm.


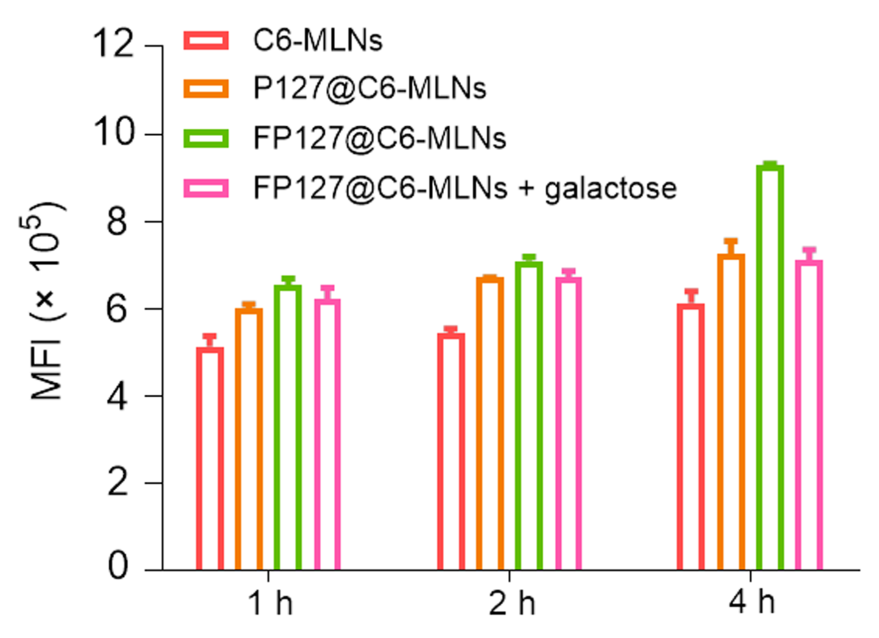


**Fig. S7.** Green fluorescence intensities of Raw 264.7 macrophages after the treatment of C6-MLNs, P127@C6-MLNs, and FP127@C6-MLNs for 1, 2, and 4 h, respectively. Data are expressed as means ± S.E.M. (n = 3).


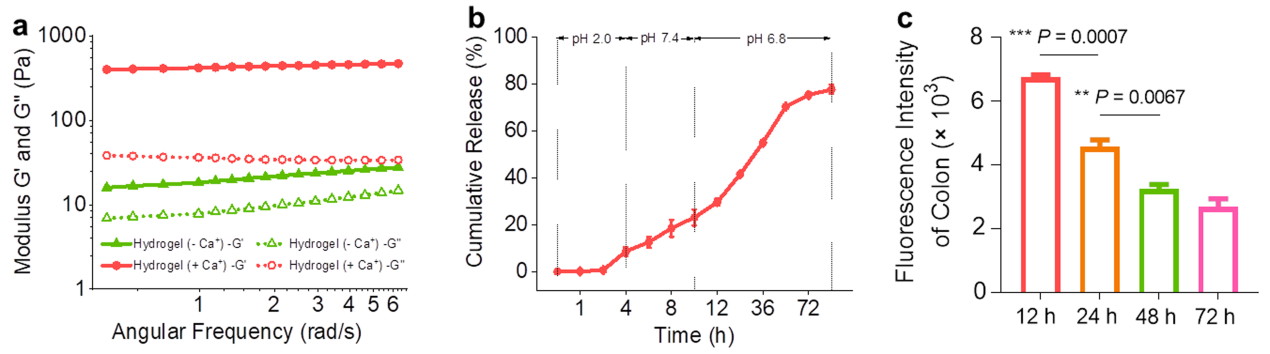


**Fig. S8.** (a) Storage modulus (G′) and loss modulus (G′′) of chitosan/alginate hydrogels with or without Ca^2+^. (b) Cumulative release behaviors of resveratrol from FP127@RN-MLN-embedded hydrogel incubated in buffers with different pH values (n = 3). (c) Quantification of fluorescence intensities of the colons from various mouse groups after oral administration of FP127@DIO-RN-MLNs for 12, 24, 48, and 72 h, respectively. Data are expressed as means ± S.E.M. (n = 3; **P* < 0.05, ***P* < 0.01, and ****P* < 0.001).


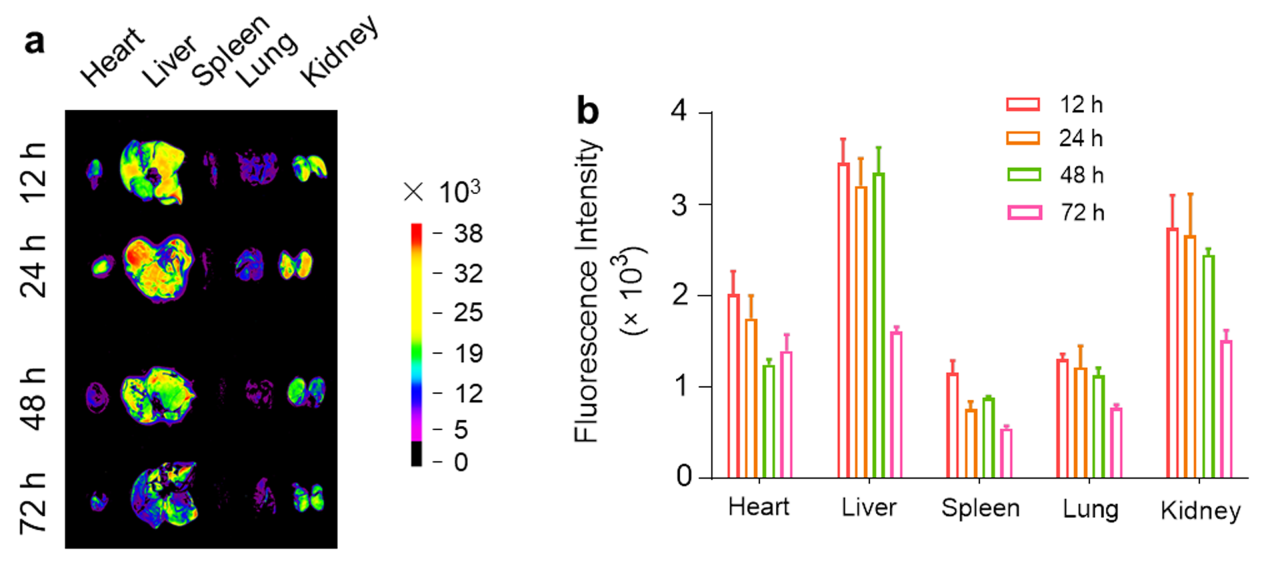


**Fig. S9.** (a) Representative fluorescence images of the five principal organs from various mouse groups after oral administration FP127@DIO-RN-MLNs for 12, 24, 48, and 72 h, respectively. (b) Quantification of fluorescence intensities of the five principal organs from various mouse groups after oral administration FP127@DIO-RN-MLNs for 12, 24, 48, and 72 h, respectively. Data are expressed as means ± S.E.M. (n = 3).


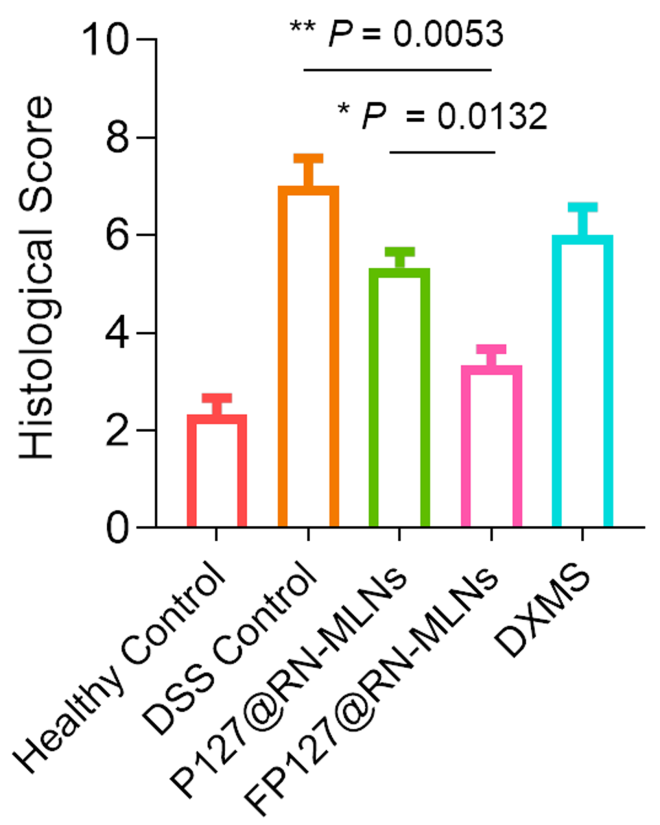


**Fig. S10.** Histological scores of the colons from various treatment groups in the UC retardation experiment. Data are expressed as means ± S.E.M. (n = 3; **P* < 0.05, ***P* < 0.01, and ****P* < 0.001).


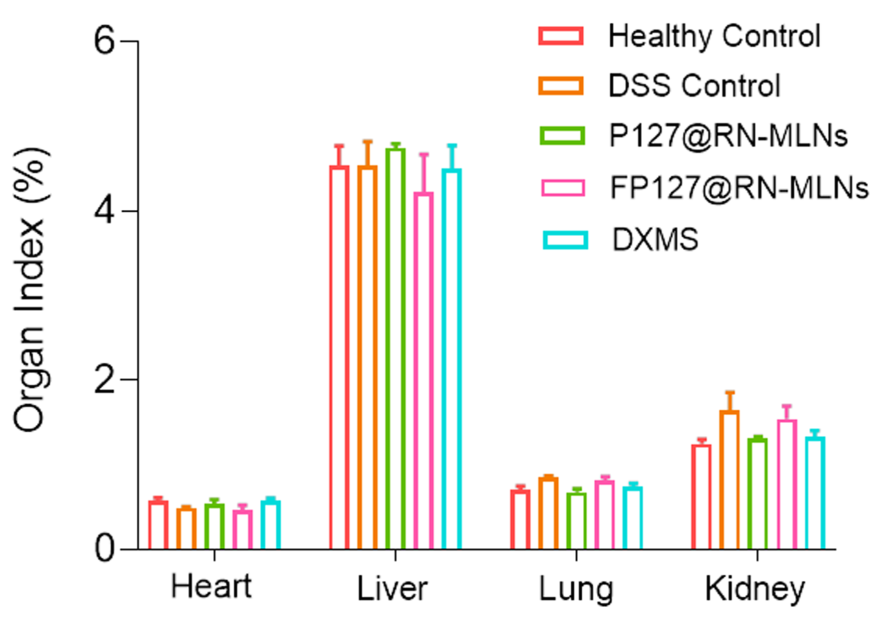


**Fig. S11.** Organ indexes of various mouse groups in the UC retardation experiment. Data are expressed as means ± S.E.M. (n = 6).


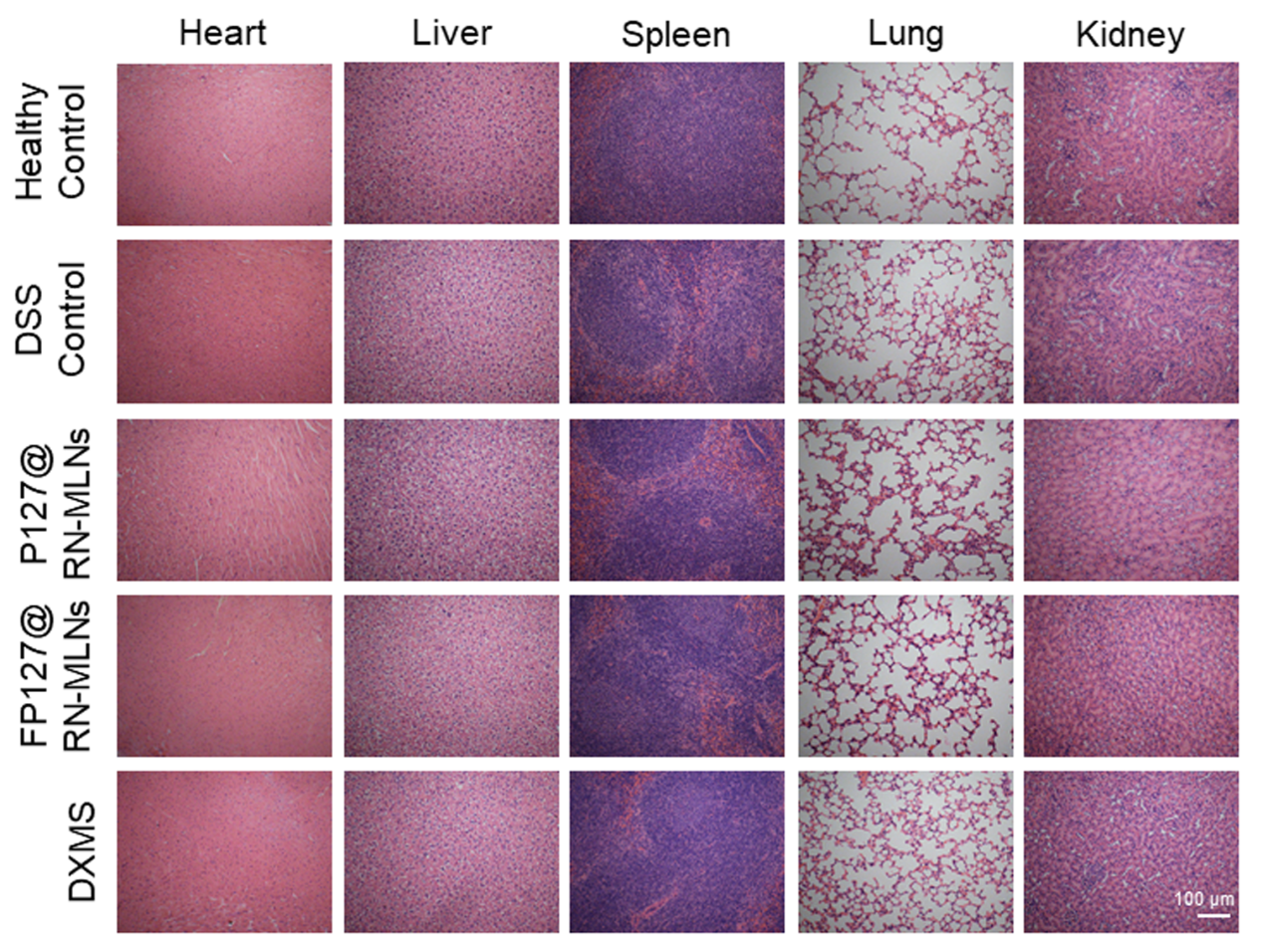


**Fig. S12.** H&E staining of the five principal organs from various treatment groups in the UC retardation experiment. Scale bar = 100 μm.


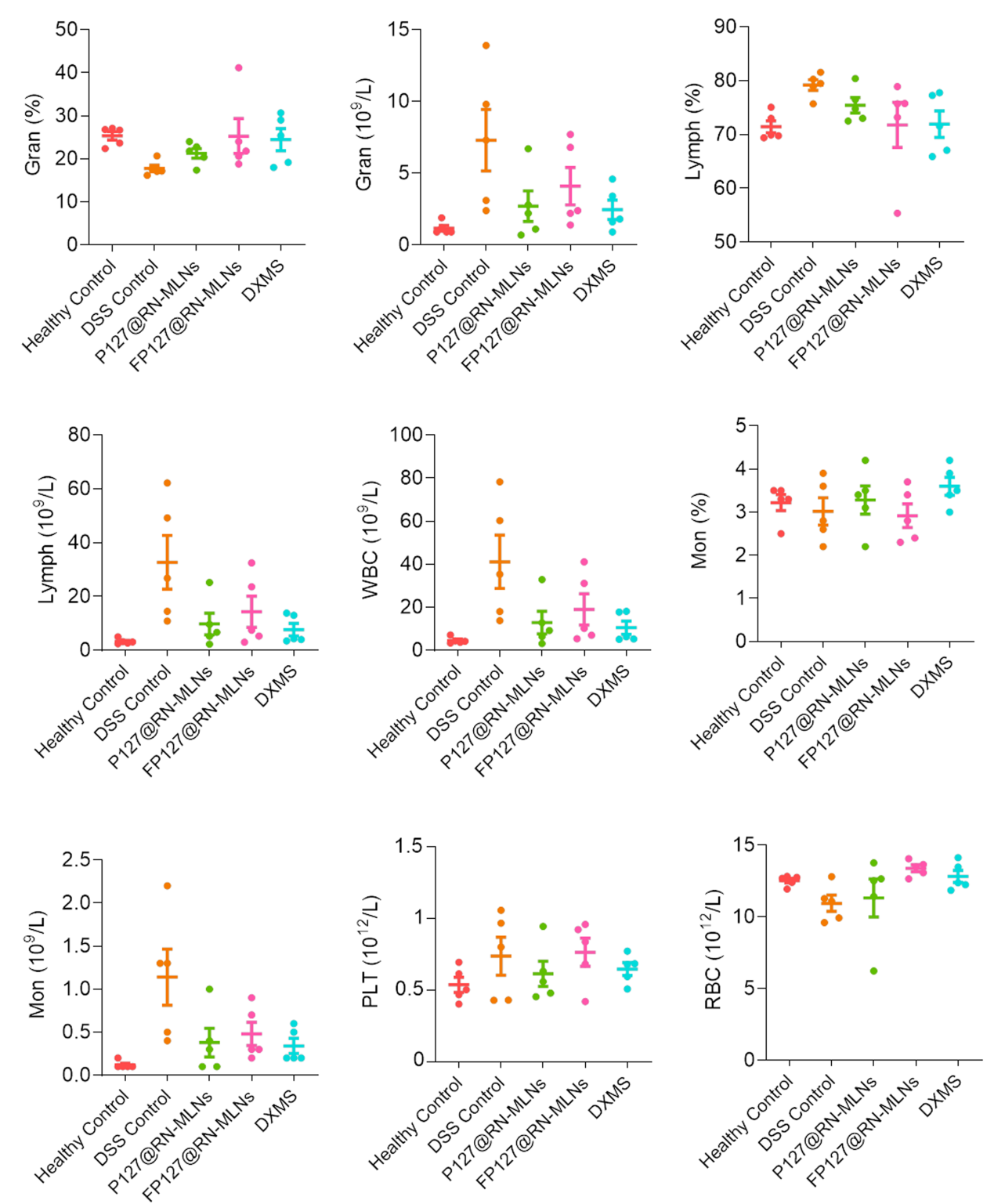


**Fig. S13.** Analysis of hematological parameters of various treatment groups in the UC retardation experiment. Data are expressed as means ± S.E.M. (n = 6).


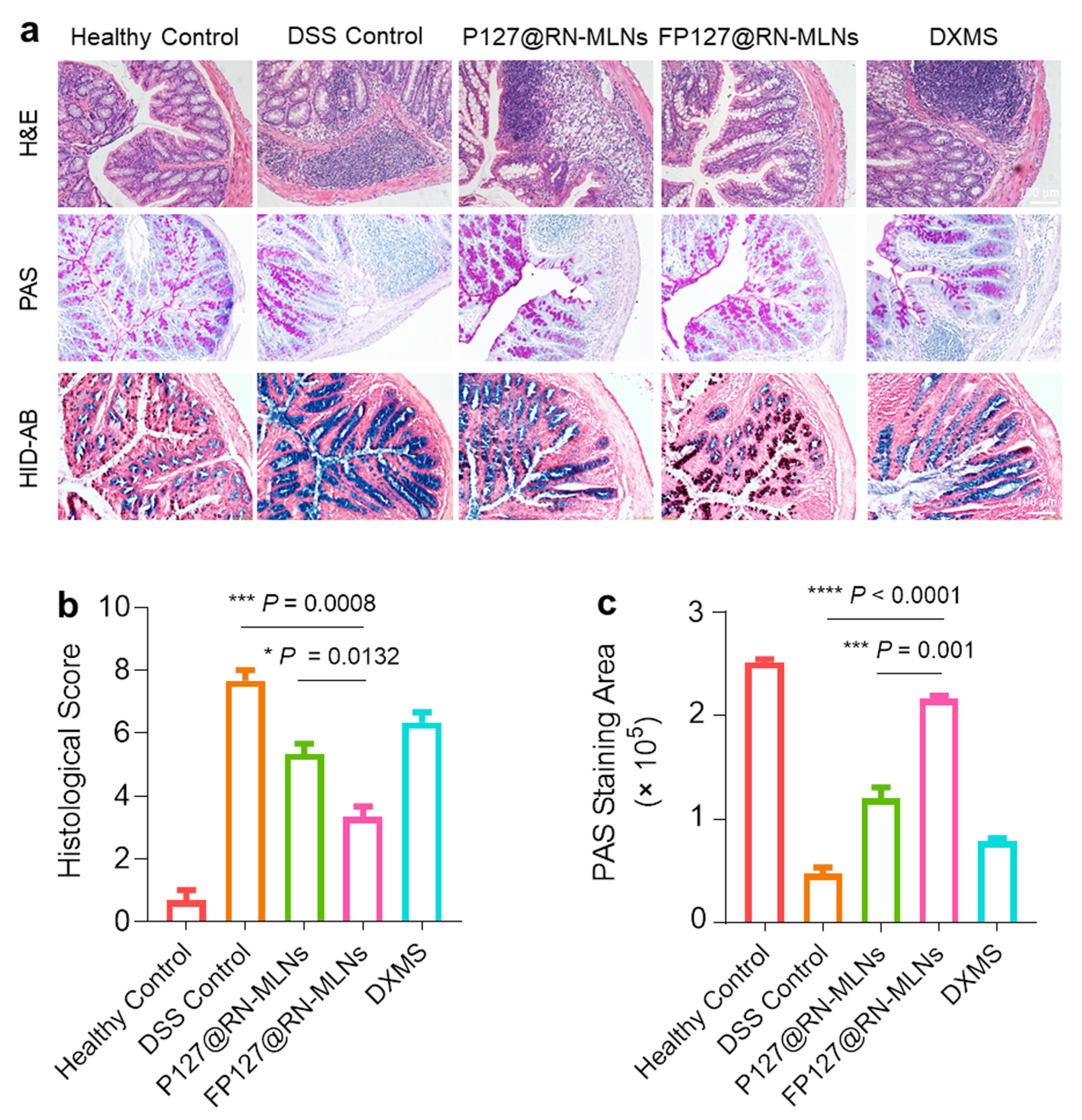


**Fig. S14.** (a) H&E, PAS, and HID-AB staining of colon sections from various treatment groups in the acute UC treatment experiment. Scale bar = 100 μm. (b) Histological scores of the colons from various treatment groups in the acute UC treatment experiment. (c) Quantifying PAS staining of colon sections from various treatment groups in the acute UC treatment experiment. Data are expressed as means ± S.E.M. (n = 3; **P* < 0.05, ***P* < 0.01, ****P* < 0.001, and *****P* < 0.0001).


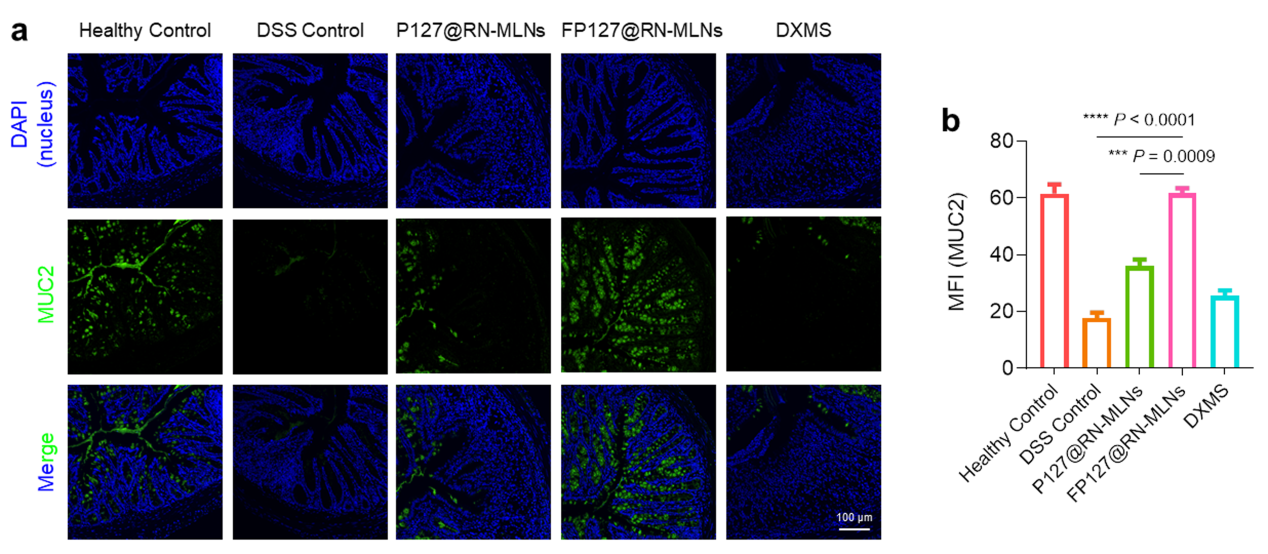


**Fig. S15.** (a) Immunofluorescence staining images of mucin (MUC2) in the colons from various treatment groups in the acute UC treatment experiment. MUC2 was stained green, and nuclei were stained blue. Scale bar = 100 μm. (b) Relative fluorescence intensities of MUC2 in the colon sections from various treatment groups in the acute UC treatment experiment. Data are expressed as means ± S.E.M. (n = 3; **P* < 0.05, ***P* < 0.01, ****P* < 0.001, and *****P* < 0.0001).


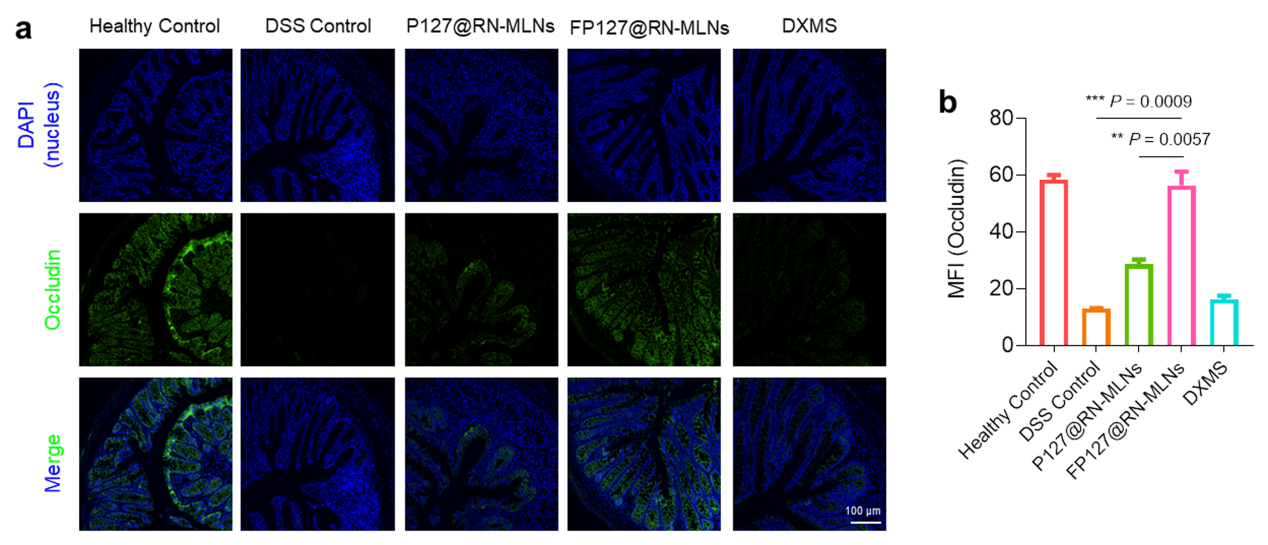


**Fig. S16.** (a) Immunofluorescence staining images of Occludin in the colons from various treatment groups in the acute UC treatment experiment. Occludin was stained green, and nuclei were stained blue. Scale bar = 100 μm. (b) Relative fluorescence intensities of Occludin in the colon sections from various treatment groups in the acute UC treatment experiment. Data are expressed as means ± S.E.M. (n = 3; **P* < 0.05, ***P* < 0.01, and ****P* < 0.001).


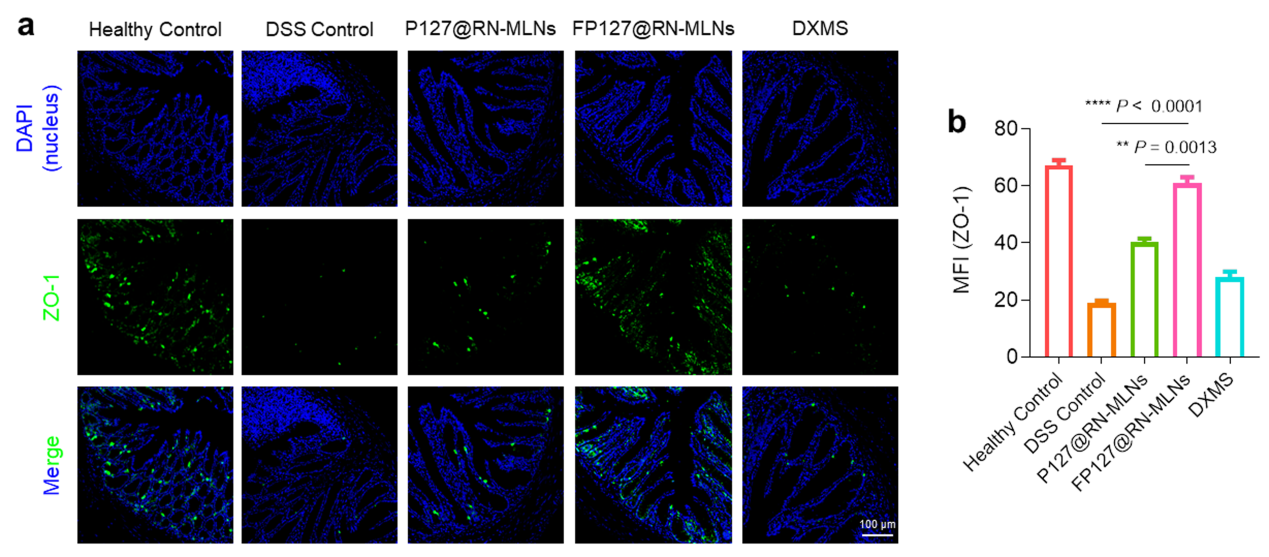


**Fig. S17.** (a) Immunofluorescence staining images of ZO-1 in the colons from various treatment groups in the acute UC treatment experiment. ZO-1 was stained green, and nuclei were stained blue. Scale bar = 100 μm. (b) Relative fluorescence intensities of ZO-1 in the colon sections from various treatment groups in the acute UC treatment experiment. Data are expressed as means ± S.E.M. (n = 3; **P* < 0.05, ***P* < 0.01, ****P* < 0.001, and *****P* < 0.0001).


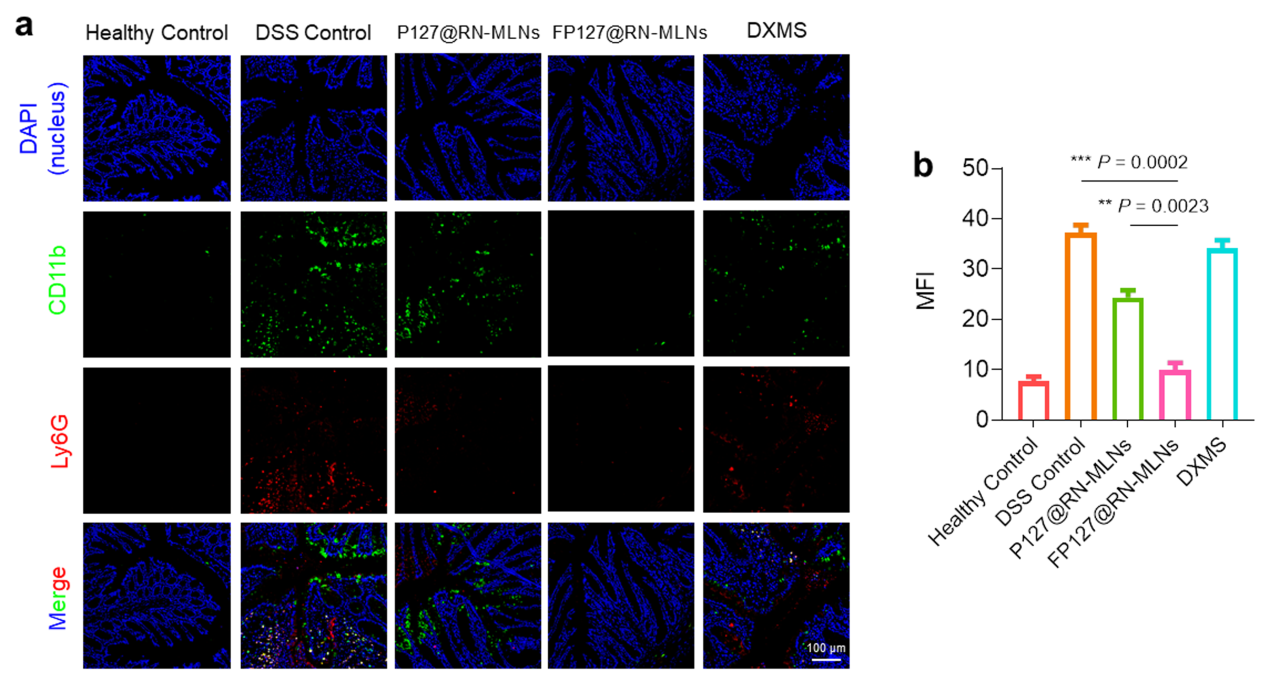


**Fig. S18.** (a) Immunofluorescence staining images of neutrophils in the colons from various treatment groups in the acute UC treatment experiment. CD11b, Ly6G, and DAPI were stained green, red, and blue, respectively. Scale bar = 100 μm. (b) Relative fluorescence intensities of Ly6G in the colon sections from various treatment groups in the acute UC treatment experiment. Data are expressed as means ± S.E.M. (n = 3; **P* < 0.05, ***P* < 0.01, and ****P* < 0.001).


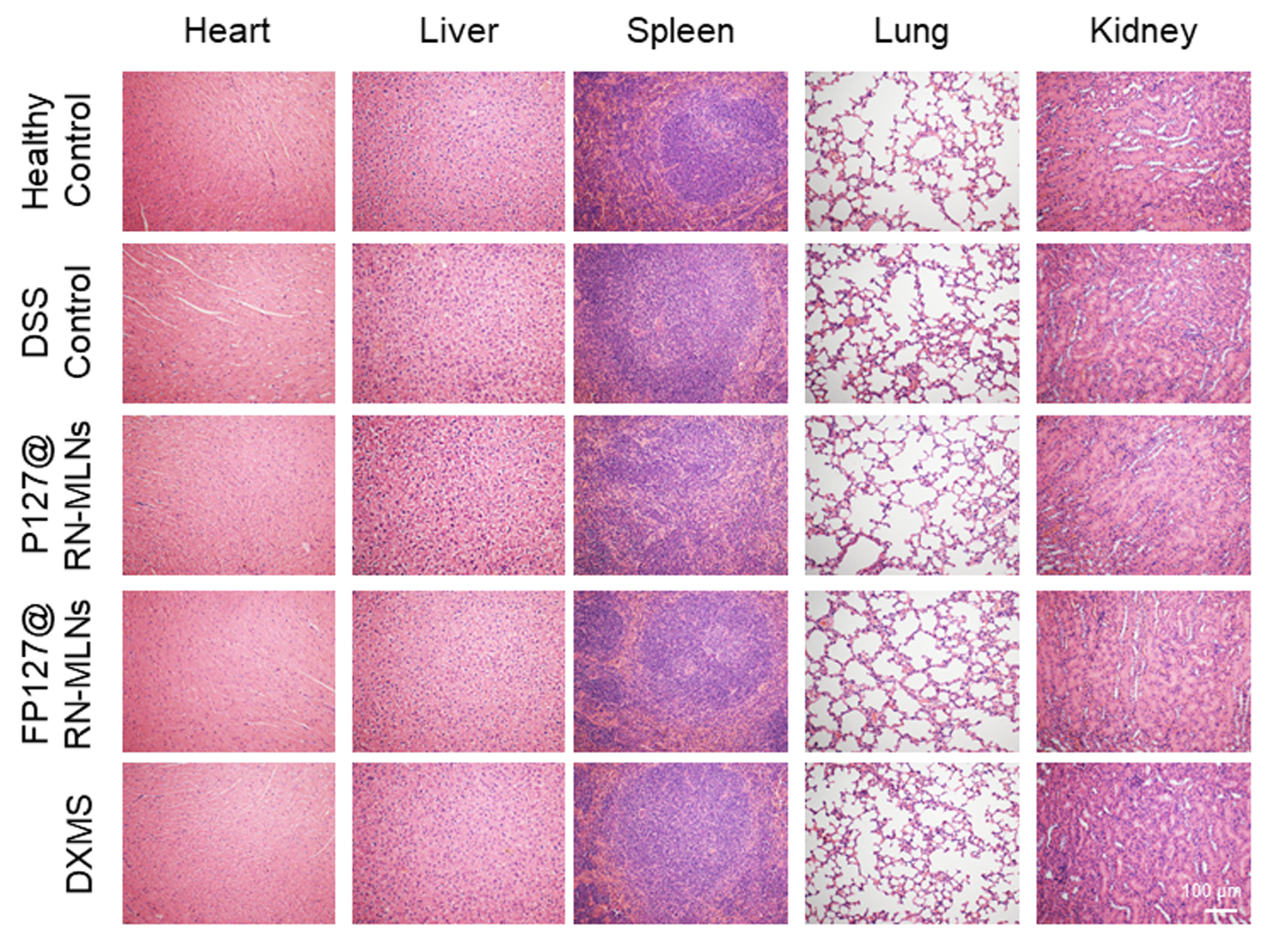


**Fig. S19.** H&E staining of the five principal organs from various treatment groups in the acute UC treatment experiment. Scale bar = 100 μm.


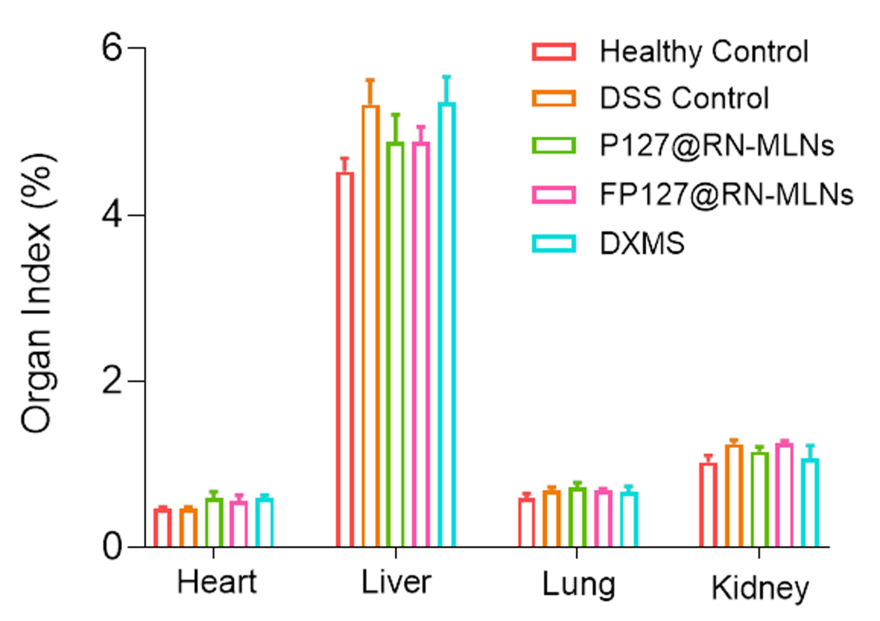


**Fig. S20.** Organ indexes of various mouse groups in the acute UC treatment experiment. Data are expressed as means ± S.E.M. (n = 6).


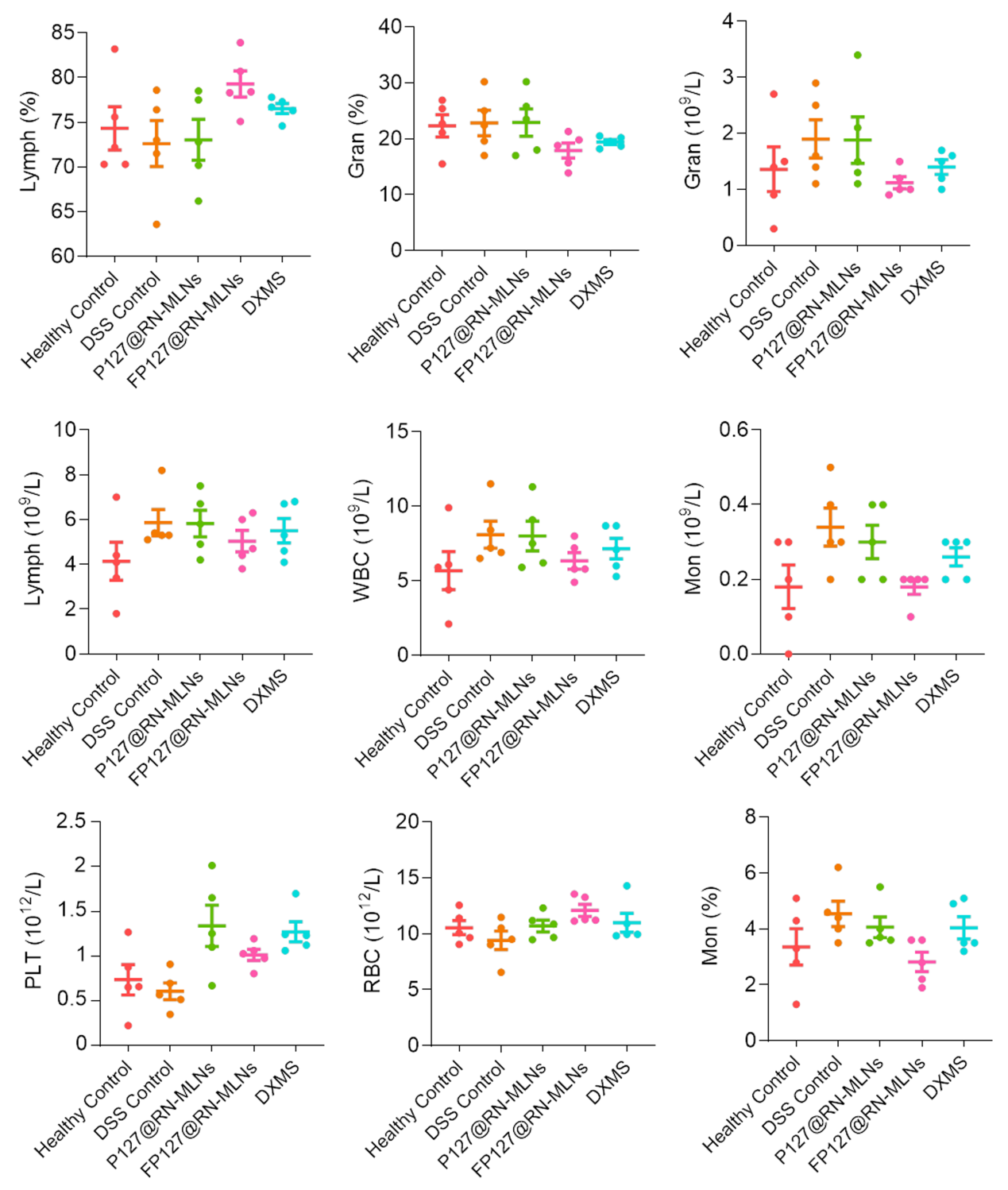


**Fig. S21.** Analysis of hematological parameters of various treatment groups in the acute UC treatment experiment. Data are expressed as means ± S.E.M. (n = 6).


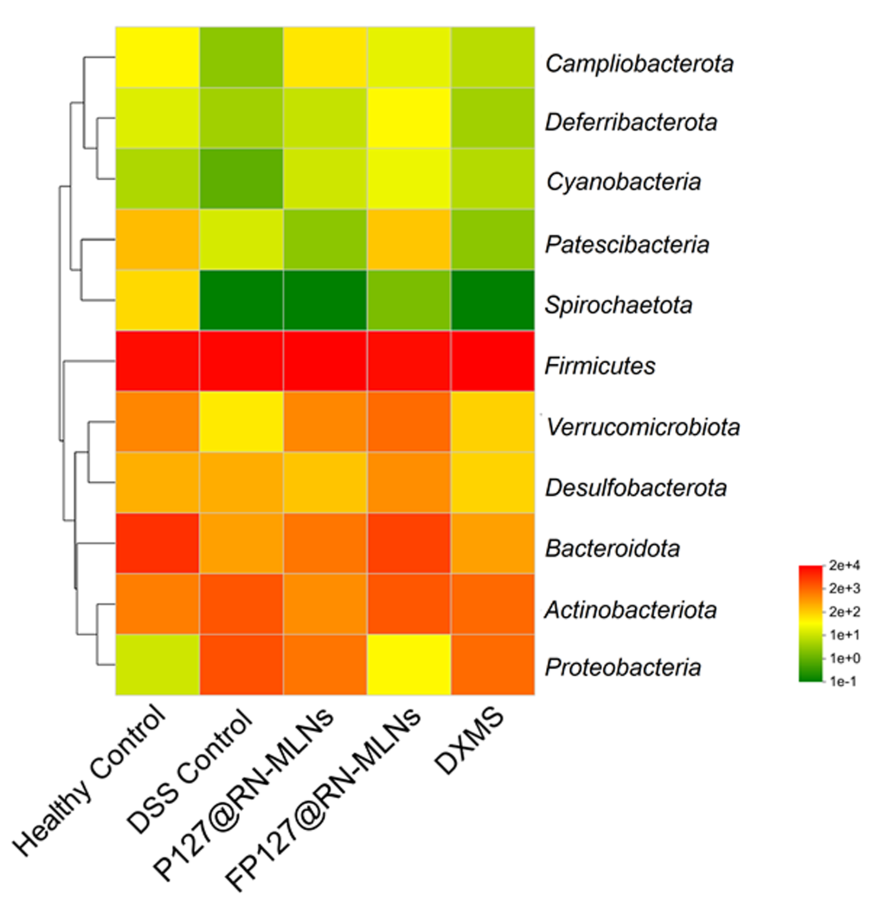


**Fig. S22.** Heat-map showing intestinal microbial cluster analysis for various treatment groups in the acute UC treatment experiment.


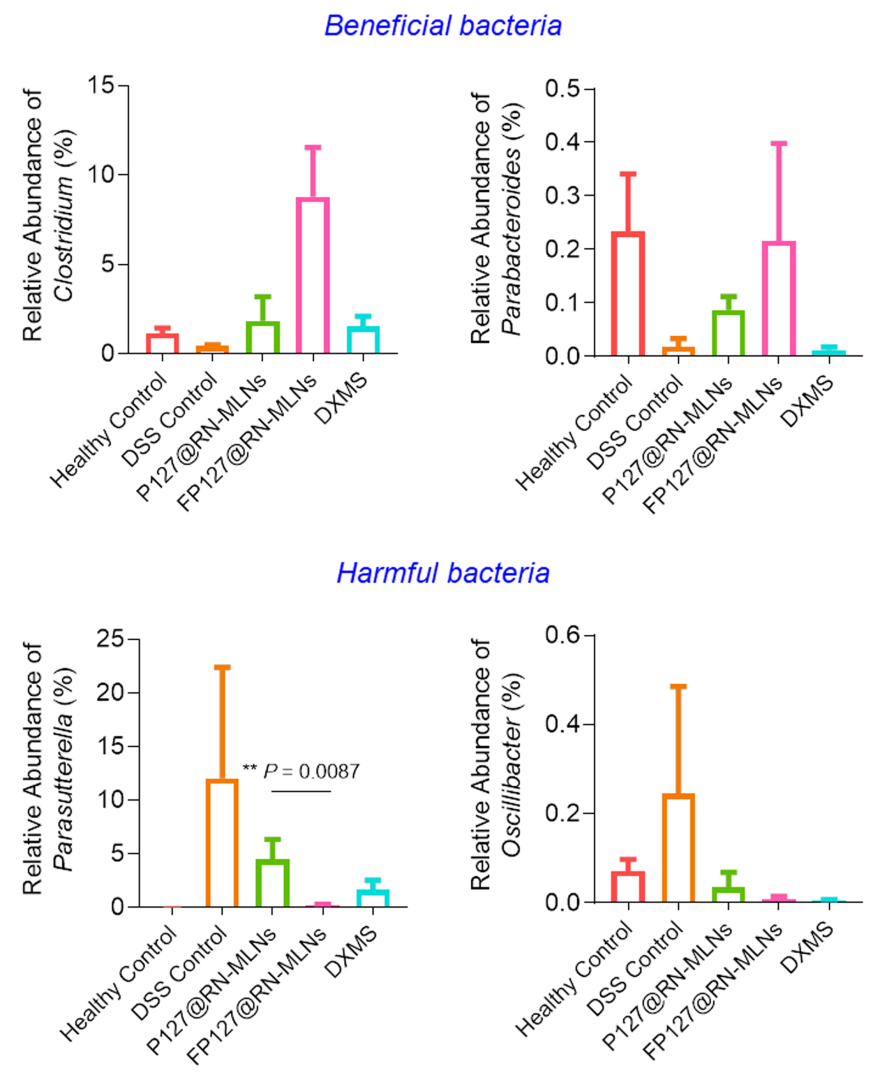


**Fig. S23.** Relative abundance of *Clostridium, Parabacteroides,* *Parasutterella*, and *Oscillibacter* in the feces from various treatment groups in the acute UC treatment experiment. Data are expressed as means ± S.E.M. (n = 3).


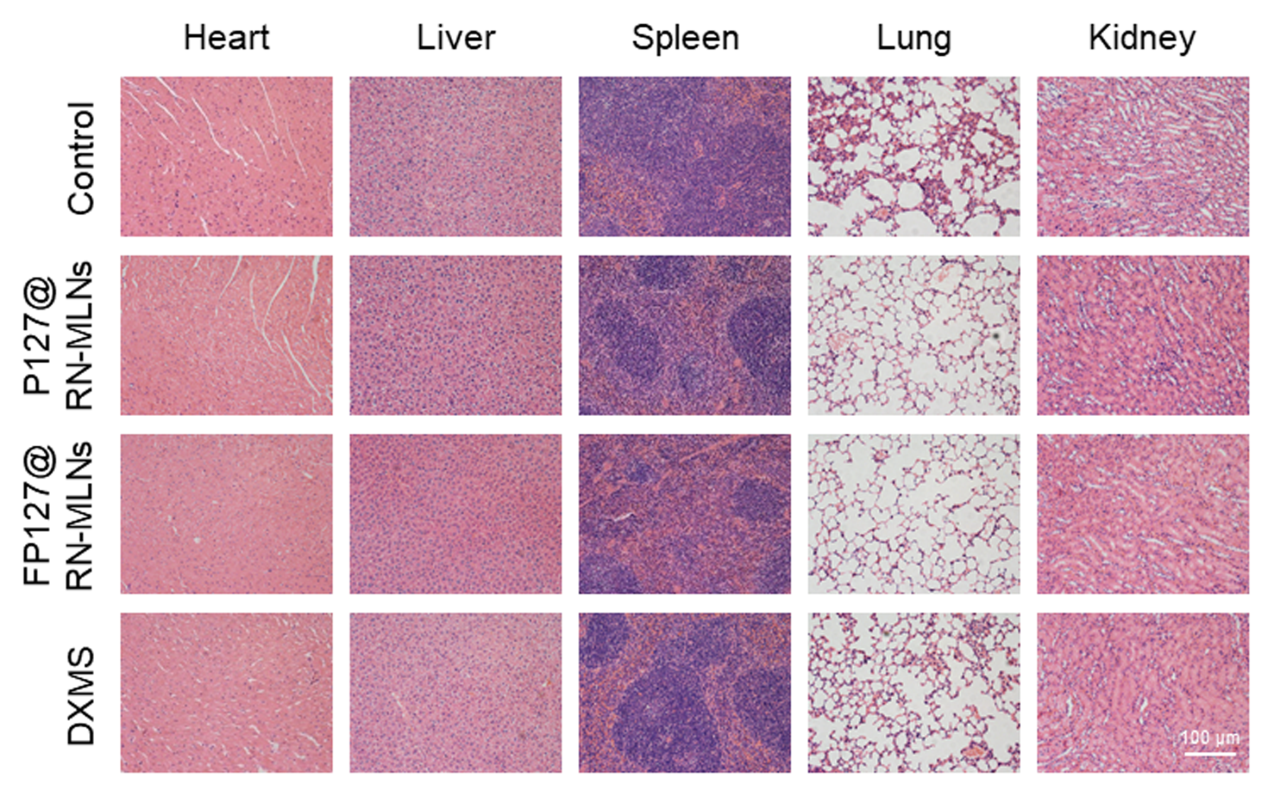


**Fig. S24.** H&E staining of the five principal organs from various treatment groups in the chronic UC treatment experiment. Scale bar = 100 μm.


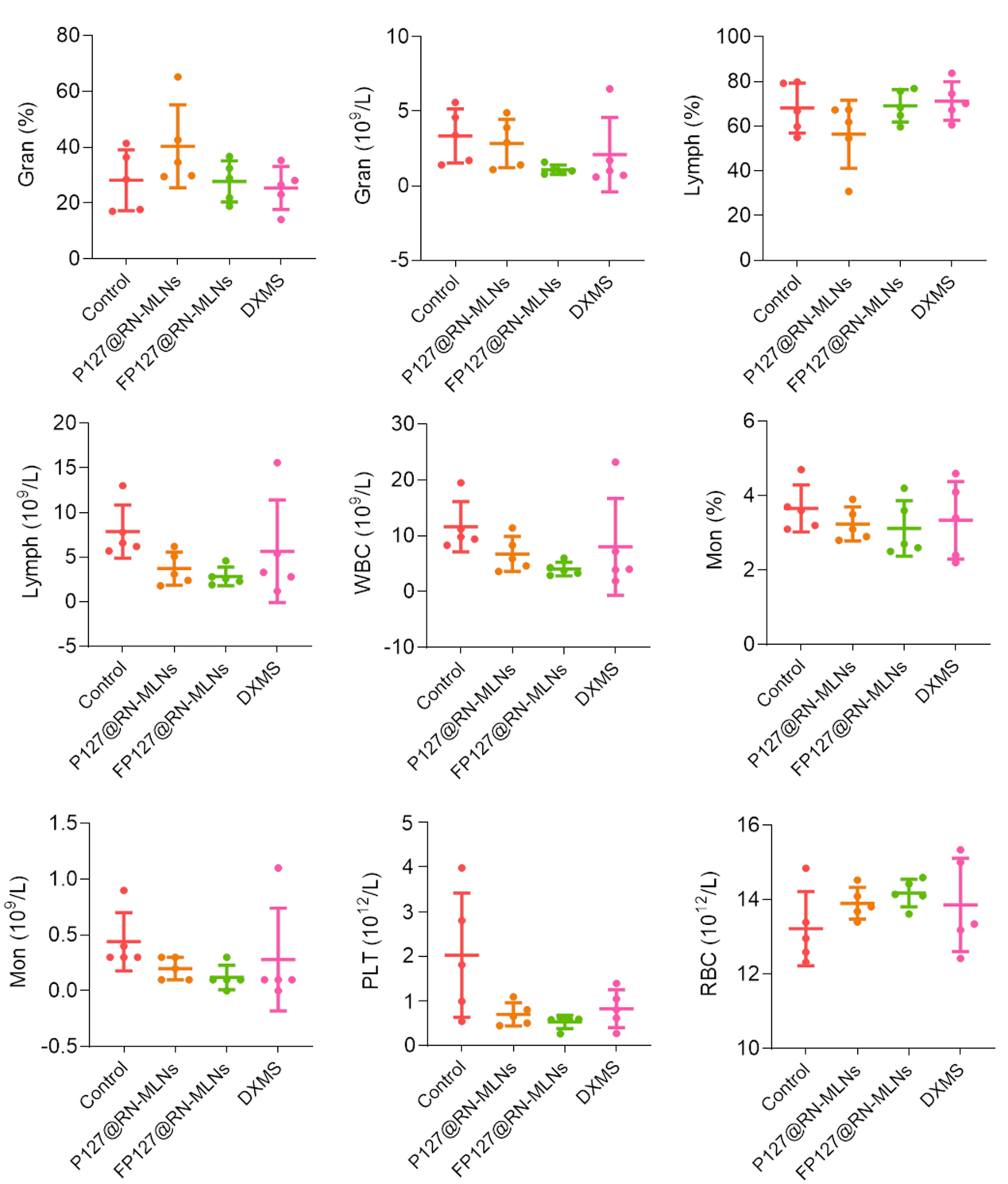


**Fig. S25.** Analysis of hematological parameters of various treatment groups in the chronic UC treatment experiment. Data are expressed as means ± S.E.M. (n = 6).

**References**

[1] Ma Y, Duan L, Sun J, Gou S, Chen F, Liang Y, Dai F, Xiao B. Oral nanotherapeutics based on Antheraea pernyi silk fibroin for synergistic treatment of ulcerative colitis. *Biomaterials.* 2022; 282 : 121410.

[2] Ma Y, Tong X, Huang Y, Zhou X, Yang C, Chen J, Dai F, Xiao B. Oral administration of hydrogel-embedding silk sericin alleviates ulcerative colitis through wound healing, anti-inflammation, and anti-oxidation. *Acs Biomater Sci Eng.* 2019; 5 (11): 6231-6242.

[3] Liu S, Cao Y, Ma L, Sun J, Ramos-Mucci L, Ma Y, Yang X, Zhu Z, Zhang J, Xiao B. Oral antimicrobial peptide-EGCG nanomedicines for synergistic treatment of ulcerative colitis. *J Control Release.* 2022; 347 : 544-560.

[4] Cao Y, Liu S, Ma Y, Ma L, Zu M, Sun J, Dai F, Duan L, Xiao B. Oral nanomotor-enabled mucus traverse and tumor penetration for targeted chemo-sono-immunotherapy against colon cancer. *Small.* 2022; 18 (42): e2203466.

[5] Xiao B, Viennois E, Chen Q, Wang L, Han M K, Zhang Y, Zhang Z, Kang Y, Wan Y, Merlin D. Silencing of intestinal glycoprotein CD98 by orally targeted nanoparticles enhances chemosensitization of colon cancer. *ACS Nano.* 2018; 12 (6): 5253-5265.
